# Palliative Care for Advanced Cancer in Medicare Advantage and Physician Networks

**DOI:** 10.1001/jamanetworkopen.2026.36865

**Published:** 2026-09-30

**Authors:** Xin Hu, Changchuan Jiang, Youngmin Kwon, Qinjin Fan, Kewei Sylvia Shi, Zhiyuan Zheng, Jingxuan Zhao, Craig Evan Pollack, Amanda L. Blackford, Joan L. Warren, Xuesong Han, K. Robin Yabroff

**Affiliations:** 1 Department of Radiation Oncology, School of Medicine, Emory University, Atlanta, Georgia; 2 Winship Cancer Institute, Emory University, Atlanta, Georgia; 3 Department of Health Policy and Management, Rollins School of Public Health, Emory University, Atlanta, Georgia; 4 Department of Internal Medicine, UT Southwestern Medical Center, Dallas, Texas; 5 Department of Health Policy, Vanderbilt University Medical Center, Nashville, Tennessee; 6 Department of Surveillance, Prevention, & Health Services Research, American Cancer Society, Atlanta, Georgia; 7 Bloomberg School of Public Health, Johns Hopkins University, Baltimore, Maryland; 8 School of Nursing, Johns Hopkins University, Baltimore, Maryland; 9 Division of Quantitative Sciences, Department of Oncology, Johns Hopkins University School of Medicine, Baltimore, Maryland

## Abstract

**Importance:**

Palliative care (PC) is guideline recommended to improve symptoms and quality of life concurrent with cancer-directed therapy for patients with advanced cancer, yet utilization remains low among Medicare beneficiaries. While Medicare Advantage (MA) enrollment has surpassed traditional Medicare (TM), how PC and other health care utilization differs between MA and TM for beneficiaries with advanced cancer is unknown.

**Objective:**

To compare PC, systemic therapy, and hospice utilization among patients with advanced cancer covered by MA vs TM and evaluate whether differences are associated with physician network composition.

**Design, Setting, and Participants:**

This retrospective cohort study used linked Surveillance, Epidemiology, and End Results (SEER) and Medicare data for beneficiaries aged 66 years or older diagnosed with distant-stage breast, colorectal, lung, pancreatic, or prostate cancer from January 1, 2016, to December 31, 2021, who had continuous MA or TM enrollment and at least 2 months’ survival. Beneficiaries were followed up from diagnosis until the earlier of death or December 31, 2021. Each beneficiary was assigned a treating oncologist; MA beneficiaries within each plan were matched 1:1 to TM beneficiaries based on treating oncologists. Data were analyzed from November 18, 2025, to July 26, 2026.

**Exposure:**

Medicare plan type.

**Main Outcomes and Measures:**

Time to first PC, systemic therapy, and hospice utilization. Multivariable models estimated the associations between MA enrollment and outcomes before and after matching on treating oncologist, adjusting for sociodemographic and clinical characteristics.

**Results:**

Among 135 402 eligible beneficiaries, 32.3% were enrolled in MA and 67.7% in TM; mean (SD) age was 76.2 (6.7) years, and 53.3% were male. Medicare Advantage beneficiaries had higher 6-month cumulative incidence of PC compared with TM beneficiaries (13.3% vs 9.3%; adjusted hazard ratio [AHR], 1.39 [95% CI, 1.35-1.44]; *P* < .001), particularly those in a health maintenance organization (HMO) plan (AHR, 1.66 [95% CI, 1.60-1.73]; *P* < .001). After matching, there were no overall differences between MA and TM in PC receipt (AHR, 1.05 [95% CI, 0.98-1.12]; *P* = .19), and the AHR was attenuated for HMO plans vs TM (AHR, 1.16 [95% CI, 1.08-1.24]; *P* < .001). Medicare Advantage beneficiaries had lower systemic therapy receipt (adjusted probability difference, −5.38 percentage points [pp]; 95% CI, −6.02 to −4.74 pp; *P* < .001) and higher hospice enrollment (3.42 pp; 95% CI, 2.82-4.03 pp; *P* < .001) than TM beneficiaries; differences persisted after matching (systemic therapy: −5.70 pp [95% CI, −6.89 to −4.52 pp]; *P* < .001; hospice enrollment: 1.68 pp [95% CI, 0.56-2.80 pp]; *P* = .003).

**Conclusions and Relevance:**

In this retrospective cohort study of Medicare beneficiaries with advanced cancer, MA enrollment was associated with increased PC utilization, primarily attributable to differential oncologist network composition. Persistent differences in systemic therapy use and hospice enrollment after matching by treating oncologists suggest that MA plan–level features beyond physician networks and patient characteristics may be associated with these outcomes.

## Key Points

**Question:**

Do palliative care (PC), systemic therapy, and hospice utilization differ between Medicare Advantage (MA) and traditional Medicare (TM) beneficiaries with advanced cancer, and is physician network composition associated with these differences?

**Findings:**

In this cohort study of 135 402 Medicare beneficiaries, MA enrollees had higher PC utilization than TM beneficiaries, but this difference was largely attenuated after matching patients to the same treating oncologists. In contrast, lower systemic therapy and higher hospice utilization among MA beneficiaries persisted after matching.

**Meaning:**

The findings suggest that differences in PC between MA and TM beneficiaries are associated with physician network composition, highlighting the importance of monitoring network adequacy and access to PC.

## Teaser

This cohort study compares receipt of palliative care, systemic therapy, and hospice enrollment between Medicare Advantage and traditional Medicare beneficiaries with advanced cancer and explores whether differences are associated with oncology network composition.

## Introduction

Palliative care (PC) is associated with improved quality of life through symptom management, communication about goals and preferences, and psychosocial support for individuals with serious illness, including advanced cancers.^1^ Clinical guidelines from the American Society of Clinical Oncology and the National Comprehensive Cancer Network (NCCN) recommend early integration of specialty outpatient PC concurrent with cancer-directed therapy—primarily systemic therapy for patients with advanced cancers.^2,3,4,5^ However, early PC remains underused: among traditional Medicare beneficiaries with common distant-stage cancers, utilization reached only about 10% by 2019,^6^ with persistent disparities across sociodemographic groups.^7,8,9,10,11^

Medicare covers nearly all adults aged 65 years or older and is the largest payer for cancer care in the US, largely because cancer incidence increases with age. Historically, most beneficiaries received coverage through traditional Medicare (TM), but enrollment in the privately operated alternative, Medicare Advantage (MA), increased substantially in the past decade and has now surpassed half of Medicare beneficiaries.^12^ Yet, evidence on PC utilization among beneficiaries with advanced cancer has focused on TM,^6,8,9^ and little is known about whether PC utilization differs in MA.

Medicare Advantage plans, paid by a risk-adjusted capitated amount per member, are designed to reduce costs through managed care principles, including utilization management and contracted physician or health care organizations, which can shape access to and patterns of cancer care.^13^ Moreover, hospice remains a carve-out benefit (ie, hospice services are covered by TM even for MA enrollees), giving MA plans financial incentives to facilitate early hospice enrollment.^14^ Studies found that MA enrollment was associated with lower likelihood of receiving systemic therapy, higher hospice enrollment, and worse survival than TM.^14,15,16,17,18,19,20^ However, to our knowledge, PC has not been evaluated as a distinct component of cancer care delivery in studies of MA vs TM, and how plan-level features contribute to differences in PC remains unknown.

Several mechanisms may drive the differences in PC between MA and TM. Because specialty PC is billed through evaluation and management codes^21^ that typically have lower cost than systemic therapies, it may be less influenced by prior authorization. Palliative care access may instead be especially sensitive to network composition, as referrals are commonly initiated by treating oncologists^7^ and timely access depends on whether PC specialists are available within the same health system.^6^ Narrow physician networks could therefore limit PC access if plans exclude oncology systems that house established specialty PC programs, and prior work has shown that many MA networks exclude National Cancer Institute (NCI)–designated comprehensive cancer centers, which typically have integrated PC programs.^22^

Network breadth is not the only structural feature that may shape PC access. Even given similar network size, integrated delivery models, which are more common among some health maintenance organization (HMO) plans, may facilitate PC through centralized care management and coordinated referral processes. The association between MA enrollment and PC use may therefore be heterogenous, with higher utilization concentrated in more integrated plans. These differences may be particularly consequential for patients with advanced-stage cancers, as PC is closely intertwined with systemic therapy decisions and hospice transitions.^23,24^

Accordingly, this study addressed 2 questions. First, do PC utilization, systemic therapy receipt, and hospice enrollment differ between MA and TM beneficiaries with distant-stage cancers, and do these differences vary across MA plan types? Second, to what extent are observed differences explained by oncologist networks? We hypothesized that higher PC utilization among MA beneficiaries would be concentrated among more integrated plan types and differences in PC would be substantially explained by oncologist network composition. In contrast, we anticipated lower systemic therapy and higher hospice enrollment among MA beneficiaries and that these differences would be less attributable to oncologist networks.

## Methods

### Study Design and Sample

In this retrospective cohort study, we used 2024 Surveillance, Epidemiology, and End Results (SEER)–Medicare linked data and included Medicare beneficiaries newly diagnosed with cancer from January 1, 2016, to December 31, 2021; fee-for-service claims through December 31, 2022; and MA encounter data through December 31, 2021.^25^ SEER data include tumor characteristics, patient demographics, and neighborhood-level measures from regions covering approximately 35% of the US population.^26^ Medicare fee-for-services claims and MA encounter data capture health care utilization for TM and MA beneficiaries, respectively. We also used the Medicare Data on Provider Practice and Specialty (MD-PPAS)^27^ to obtain physician information. This study was approved by the Emory University institutional review board under expedited review, with an informed consent waiver for secondary data analysis. The study followed the Strengthening the Reporting of Observational Studies in Epidemiology (STROBE) reporting guideline.

We identified patients with de novo distant-stage breast cancer, non–small cell lung cancer (NSCLC), small cell lung cancer (SCLC), colorectal cancer, prostate cancer, or pancreas cancer from January 1, 2016, to December 31, 2021, based on *International Classification of Diseases for Oncology, 3rd Edition (ICD-O-3)* and histology codes (eTable 1 in Supplement 1).^28^ For patients with multiple distant-stage diagnoses, we selected the first qualifying diagnosis. Inclusion criteria were diagnosis at or later than age 66 years, at least 2 months’ survival after diagnosis, and continuous enrollment in Medicare Parts A and B from 12 months before cancer diagnosis through death or end of follow-up (December 31, 2021). Beneficiaries who switched between TM and MA during follow-up or were diagnosed at autopsy or on the death certificate were excluded (eFigure 1 in Supplement 1). We also defined 3 subcohorts: decedents who died by December 31, 2021, to evaluate hospice enrollment; beneficiaries with additional Medicare Part D coverage to evaluate systemic therapy receipt (2 outcomes intertwined with PC and known to differ between TM and MA^14,15,16,17,18,19^); and beneficiaries surviving 6 months or more to address prognostic selection bias.

To assess the extent to which oncologist networks contributed to differences in PC and related outcomes between MA and TM, we used a shared-physician design. Prior studies have implemented such designs by matching patients to common physicians and incorporating physician fixed effects.^29,30^ Following our team’s previous approach,^6,31^ our primary analysis implemented physician-based matching. We first assigned each beneficiary a treating oncologist, defined as the National Provider Identifier (NPI) that appeared most frequently on outpatient or carrier claims with cancer diagnosis codes from 90 days before through 180 days after diagnosis, death, or end of follow-up. Because a nontrivial share of MA claims lacked individual NPIs, we used the MD-PPAS file to recover individual NPIs and reduce missingness by approximately 10% (eTable 2 and eMethods 1 in Supplement 1). For each MA plan, we performed 1:1 exact matching of MA to TM beneficiaries by treating oncologist using a greedy algorithm, which selects the control unit that best matches the treated unit without replacement; thus, a TM beneficiary could be matched to more than 1 MA plan because we conducted matching separately for each MA plan. We did not match additional patient characteristics because it would substantially reduce successful matches for MA contracts with a small number of beneficiaries. We therefore adjusted for patient covariates (age, sex, race and ethnicity, metropolitan residence, marital status, dual eligibility, Yost Index quintile, cancer site, year of diagnosis, and NCI Comorbidity Index) in regressions using both the unmatched and matched samples. Matching was conducted separately for the primary cohort and each subcohort.

### Measures

#### Time to First Palliative Care Visit

Our primary outcome was time to first outpatient PC visit, identified by claims with corresponding diagnosis codes or physician specialty codes^8,32^ through 6 months after cancer diagnosis or until hospice admission or death, whichever occurred first. A recent validation study demonstrated that PC diagnosis codes with specialty codes have 78.1% sensitivity and nearly 100% specificity for identifying outpatient PC.^33^

#### Systemic Therapy Use and Hospice Enrollment Among Decedents

Systemic therapy receipt was defined as at least 1 claim containing a qualifying antineoplastic drug or infusion procedure code from diagnosis to death: *International Classification of Diseases, Ninth Revision* or *International Statistical Classification of Diseases and Related Health Problems, Tenth Revision* procedure code, Healthcare Common Procedure Coding System code, or a generic or brand name oral agent (eTable 3 in Supplement 1).^6^ The eligible drug list was reviewed by oncology clinician members of the study team (C.J.) and restricted to therapies with US Food and Drug Administration–approved cancer indications and NCCN guideline recommendations. Hospice enrollment was identified by any hospice claim.

#### Study Covariates

Patient characteristics included age, sex, race and ethnicity, metropolitan residency, census-tract socioeconomic status (Yost Index^34^), dual Medicaid-Medicare eligibility, cancer type, diagnosis year, and NCI Comorbidity Index score^35^ measured 12 months before diagnosis. Race and ethnicity were included because they capture structural and social factors that are associated with both differential enrollment into Medicare Advantage and unequal access to palliative care; they were abstracted from medical records by cancer registrars at the time of diagnosis as Hispanic, non-Hispanic Black, non-Hispanic White, non-Hispanic other (American Indian or Alaska Native, Asian or Other Pacific Islander, and other unspecified race), or unknown. To mitigate biases in comorbidity burden between MA and TM due to coding intensity,^36,37^ we excluded MA claims flagged as medical record review without procedure codes when constructing comorbidity measures for MA beneficiaries. We also measured MA plan types, including HMOs, local preferred provider organizations (PPOs), regional PPOs, point of service (POS), and others.

### Statistical Analysis

We compared sample characteristics between MA and TM in the full and matched cohorts and assessed differences between matched and unmatched MA beneficiaries. We estimated the cumulative incidence of first PC encounter by MA vs TM status in both the unmatched cohort and the matched cohort. We then conducted multivariable regression analyses using the unmatched and matched samples; each set of the model included a binary indicator for MA (vs TM) and patient sociodemographic and clinical characteristics to account for potential patient selection into MA plans. We intentionally did not use instrumental variable analysis designed to address unobserved confounding; rather, we tested a specific, observable mechanism (ie, oncologist network as captured through treating oncologist matching) hypothesized to be associated with differences between MA and TM. Because TM beneficiaries could be matched to multiple MA beneficiaries, we clustered SEs at the beneficiary level in the matched cohort analysis.

Cox proportional hazards regression models accounting for death as a competing risk were used for time to first PC visit. Logistic regression with marginal effects was used for systemic therapy receipt and hospice enrollment, with estimates interpreted as adjusted probability differences.^38^ In addition to the comparison of MA and TM overall, we examined more granular MA plan types given that certain plan types, such as HMOs, have more selective contracting and limited network breadth.^39^

We performed several sensitivity analyses. First, we repeated analyses among beneficiaries with 6 or more months of survival and those who were diagnosed and died outside the COVID-19 disruption period (March 1 to December 31, 2020). Second, we stratified the analyses by cancer sites, considering different prognoses that may affect study outcomes. Third, because earlier MA encounter data completeness varied across MA contracts^40,41,42^ and our own investigation identified nonnegligible and nonrandom plan-level missingness of individual NPIs (eFigure 2 in Supplement 1), we repeated analyses restricting to MA contracts with high data completeness and no contracts with 100% missing individual NPIs, for which we expected more complete capture of PC visits. Fourth, because MA beneficiaries still showed higher mean comorbidity indices than TM beneficiaries after excluding reviewed medical records, we repeated regression analyses without the NCI Comorbidity Index. Fifth, to address concerns about sample representativeness after matching, we implemented an alternative shared-physician design that compared differences between MA and TM in outcomes with and without controlling for oncologist fixed effects (eMethods 2 in Supplement 1).

Data analyses were performed from November 18, 2025, to July 26, 2026, using SAS, version 9.4 (SAS Institute Inc) and Stata, version 18.0 (StataCorp LLC). Statistical significance was evaluated at a *P* < .05 threshold using 2-sided tests.

## Results

Among 135 402 Medicare beneficiaries with advanced cancer, 43 774 (32.3%) were enrolled in MA and 91 628 (67.7%) in TM. Mean (SD) age was 76.2 (6.7) years; 46.7% were female, and 53.3% were male. A total of 9.5% were Black; 7.3%, Hispanic; 78.2%, White; 4.8%, non-Hispanic other race; and 0.2%, unknown race and ethnicity. Medicare Advantage beneficiaries vs TM beneficiaries were more likely to be racial and ethnic minority individuals (eg, Black: 13.2% vs 7.7%; Hispanic: 11.0% vs 5.6%; *P* < .001 for both) and to reside in metropolitan areas (91.9% vs 83.7%; *P* < .001) and areas with lower socioeconomic status (Yost Index quintile 1, indicating lowest socioeconomic status: 14.9% vs 12.4%; *P* < .001) (Table). After matching by treating oncologists, 23 033 MA observations and 23 033 TM observations among 17 606 unique TM beneficiaries were included. Matched MA patients were less likely than unmatched MA patients to enroll in HMO plans (49.1% vs 76.7%; *P* < .001), with most unmatched beneficiaries enrolled in the Kaiser Foundation Health Plan (7684 of 7969 [96.4%]) (eTable 4 in Supplement 1).

**Table.** Sample Characteristics by Medicare Advantage and Traditional Medicare Before and After 1:1 Exact Matching by Treating Oncologist

Characteristic	Beneficiaries before matching, No. (%)				Observations after matching		
	Total (N = 135 402)	MA (n = 43 774 [32.3%])	TM (n = 91 628 [67.7%])	*P* value	MA (n = 23 033 [50.0%])	TM (n = 23 033 [50.0%])	*P* value
Year of diagnosis							
2016	21 931 (16.2)	5748 (13.1)	16 183 (17.7)	<.001	2936 (12.8)	2936 (12.8)	>.99
2017	22 243 (16.4)	6146 (14.0)	16 097 (17.6)		3090 (13.4)	3090 (13.4)	
2018	23 006 (17.0)	6864 (15.7)	16 142 (17.6)		3623 (15.7)	3623 (15.7)	
2019	24 377 (18.0)	8182 (18.7)	16 195 (17.7)		4336 (18.8)	4336 (18.8)	
2020	22 691 (16.8)	8225 (18.8)	14 466 (15.8)		4493 (19.5)	4493 (19.5)	
2021	21 154 (15.6)	8609 (19.7)	12 545 (13.7)		4555 (19.8)	4555 (19.8)	
Age at diagnosis, mean (SD), y	76.2 (6.7)	76.3 (6.6)	76.2 (6.7)	<.001	75.7 (6.4)	75.6 (6.5)	<.001
Age group at diagnosis, y							
66-74	61 390 (45.3)	19 641 (44.9)	41 749 (45.6)	<.001	10 977 (47.7)	11 240 (48.8)	.03
75-84	55 028 (40.6)	18 190 (41.6)	36 838 (40.2)		9414 (40.9)	9146 (39.7)	
≥85	18 984 (14.0)	5943 (13.6)	13 041 (14.2)		2642 (11.5)	2647 (11.5)	
Sex							
Female	63 220 (46.7)	20 378 (46.6)	42 842 (46.8)	.48	11 089 (48.1)	10 969 (47.6)	.26
Male	72 182 (53.3)	23 396 (53.4)	48 786 (53.2)		11 944 (51.9)	12 064 (52.4)	
Race and ethnicity							
Hispanic	9936 (7.3)	4820 (11.0)	5116 (5.6)	<.001	1995 (8.7)	1264 (5.5)	<.001
Non-Hispanic Black	12 814 (9.5)	5787 (13.2)	7027 (7.7)		2858 (12.4)	1954 (8.5)	
Non-Hispanic White	105 950 (78.2)	30 491 (69.7)	75 459 (82.4)		17 183 (74.6)	18 781 (81.5)	
Non-Hispanic other^a^	6452 (4.8)	2599 (5.9)	3853 (4.2)		965 (4.2)	983 (4.3)	
Unknown	250 (0.2)	77 (0.2)	173 (0.2)		32 (0.1)	51 (0.2)	
Metropolitan residence status^b^							
Nonmetropolitan	18 491 (13.7)	3535 (8.1)	14 956 (16.3)	<.001	2489 (10.8)	3236 (14.1)	<.001
Metropolitan	11 6911 (86.3)	40 239 (91.9)	76 672 (83.7)		20 544 (89.2)	19 797 (86.0)	
Dual eligibility^c^							
No	118 443 (87.5)	37 940 (86.7)	80 503 (87.9)	<.001	20 026 (86.9)	20 321 (88.2)	<.001
Yes	16 959 (12.5)	5834 (13.3)	11 125 (12.1)		3007 (13.1)	2712 (11.8)	
Marital status							
Married	55 218 (40.8)	18 786 (42.9)	36 432 (39.8)	<.001	9385 (40.8)	9528 (41.4)	.40
Not married	46 648 (34.5)	15 806 (36.1)	30 842 (33.7)		7917 (34.4)	7834 (34.0)	
Unknown	33 536 (24.8)	9182 (21.0)	24 354 (26.6)		5731 (24.9)	5671 (24.6)	
Yost Index quintile^d^							
1 (Lowest SES)	17 899 (13.2)	6543 (14.9)	11 356 (12.4)	<.001	3502 (15.2)	3004 (13.0)	<.001
2	20 134 (14.9)	6706 (15.3)	13 428 (14.7)		3660 (15.9)	3510 (15.2)	
3	23 091 (17.1)	7837 (17.9)	15 254 (16.6)		4112 (17.9)	4194 (18.2)	
4	27 019 (20.0)	9325 (21.3)	17 694 (19.3)		4794 (20.8)	4626 (20.1)	
5 (Highest SES)	33 038 (24.4)	10 076 (23.0)	22 962 (25.1)		4655 (20.2)	5434 (23.6)	
Unknown	14 221 (10.5)	3287 (7.5)	10 934 (11.9)		2310 (10.0)	2265 (9.8)	
Cancer site							
Breast	9237 (6.8)	2815 (6.4)	6422 (7.0)	<.001	1391 (6.0)	1674 (7.3)	<.001
Colorectal	18 861 (13.9)	5908 (13.5)	12 953 (14.1)		3063 (13.3)	3327 (14.4)	
NSCLC	59 976 (44.3)	19 567 (44.7)	40 409 (44.1)		10 708 (46.5)	10 243 (44.5)	
Pancreas	16 837 (12.4)	5509 (12.6)	11 328 (12.4)		3028 (13.2)	2889 (12.5)	
Prostate	17 520 (12.9)	5852 (13.4)	11 668 (12.7)		2255 (9.8)	2416 (10.5)	
SCLC	12 971 (9.6)	4123 (9.4)	8848 (9.7)		2588 (11.2)	2484 (10.8)	
NCI Comorbidity Index, mean (SD)^e^	0.54 (0.60)	0.58 (0.60)	0.53 (0.60)	<.001	0.56 (0.59)	0.58 (0.58)	<.001
MA plan type							
HMO	27 217 (20.1)	27 217 (62.2)	NA	NA	11 315 (49.1)	NA	NA
Local PPO	11 705 (8.6)	11 705 (26.7)	NA		8490 (36.9)	NA	
Regional PPO	1989 (1.5)	1989 (4.5)	NA		1455 (6.3)	NA	
POS	1623 (1.2)	1623 (3.7)	NA		1004 (4.4)	NA	
Other or unknown	1240 (0.9)	1240 (2.8)	NA		769 (3.3)	NA	

Abbreviations: HMO, health maintenance organization; MA, Medicare Advantage; NA, not applicable; NCI, National Cancer Institute; NSCLC, non–small cell lung cancer; POS, point of service; PPO, preferred provider organization; SCLC, small cell lung cancer; SES, socioeconomic status; TM, traditional Medicare.

^a^ Other includes American Indian or Alaska Native, Asian or Other Pacific Islander, and other unspecified race.

^b^ Based on 2013 Rural-Urban Commuting Area Codes.

^c^ With state buy-in (Medicaid) from cancer diagnosis to the end of follow-up.

^d^ The Yost Index is a composite SES score for census tracts based on median household income, median house value, median rent, percentage of residents below 150% of the federal poverty level, educational level, percentage of residents who are working class, and percentage of residents who are unemployed.

^e^ Total score range, 0 to 5.4, with higher scores indicating higher comorbidity burden.

### Palliative Care

Prior to matching, the 6-month cumulative incidence of PC was 13.3% and 9.3% among MA and TM beneficiaries, respectively (Figure 1A). In adjusted analyses, MA enrollment was associated with a higher rate of PC receipt (adjusted hazard ratio [AHR], 1.39; 95% CI, 1.35-1.44; *P* < .001). Across MA plan types, the difference between MA and TM was most pronounced in HMOs (AHR, 1.66; 95% CI, 1.60-1.73; *P* < .001), but there were no differences for local PPO, regional PPO, POS, or other plan types (Figure 2 and eTables 5 and 6 in Supplement 1).

**Figure 1.**
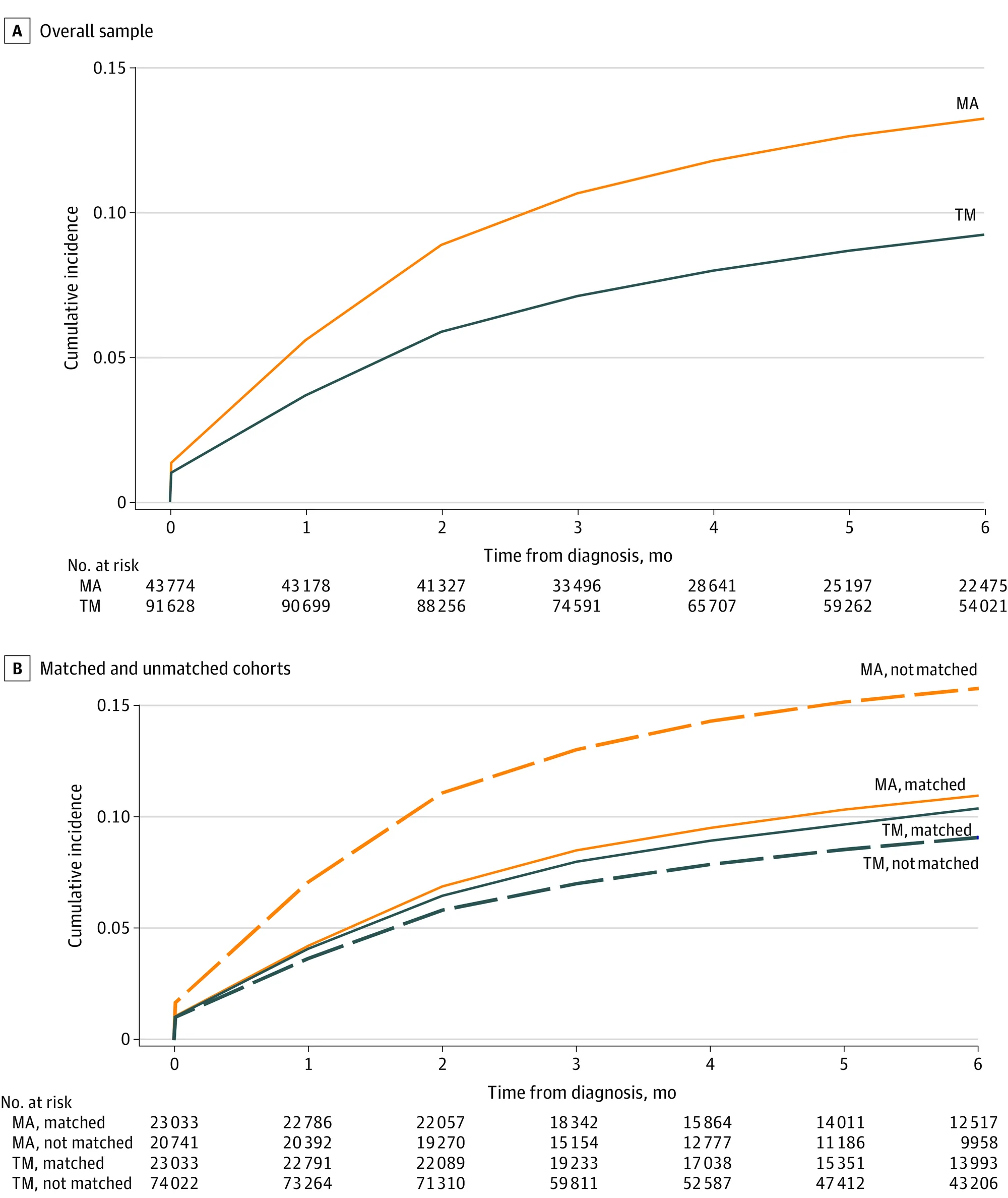
Cumulative Incidence Curves of Palliative Care Receipt Up to 6 Months After Cancer Diagnosis MA indicates Medicare Advantage; TM, traditional Medicare.

**Figure 2.**
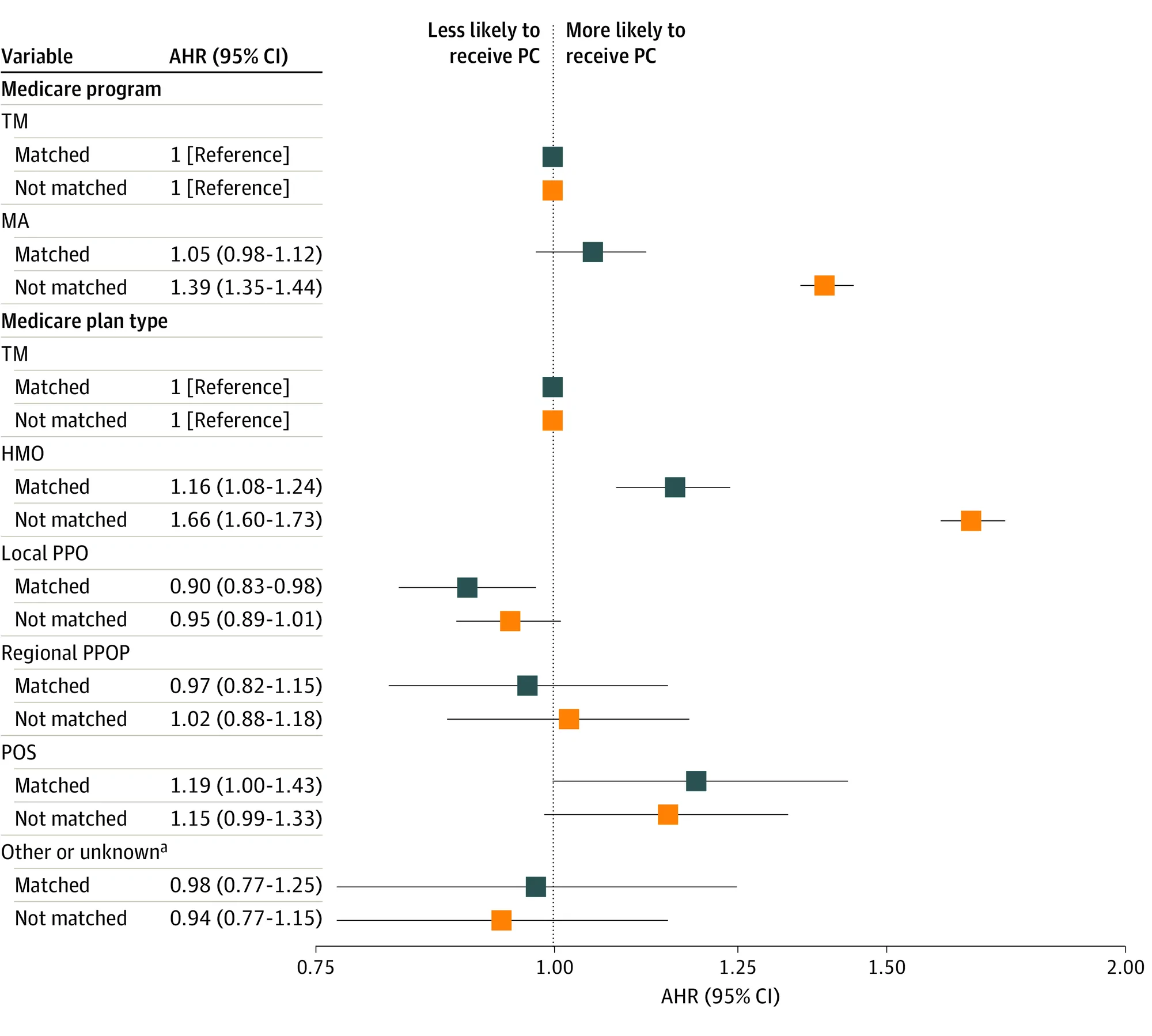
Dot-and-Whisker Plot of Adjusted Differences in Palliative Care (PC) Receipt Up to 6 Months Before and After Matching Based on Treating Oncologists Exact 1:1 matching of Medicare Advantage (MA) beneficiaries under each contract to traditional Medicare (TM) beneficiaries treated by the same oncologist was done. The sample size without matching was 135 402 and with matching was 46 066. The regression model adjusted for patient age, sex, race and ethnicity, metropolitan residence, marital status, dual eligibility, Yost Index quintile, cancer site, year of diagnosis, and National Cancer Institute Comorbidity Index. SEs were clustered to the beneficiary level in the matched cohort because some TM beneficiaries were matched to multiple MA beneficiaries. Whiskers represent 95% CIs. AHR indicates adjusted hazard ratio; HMO, health maintenance organization; POS, point of service; PPO, preferred provider organization. ^a^Other plan types included medical savings account, private fee-for-service plan, Section 1876 Cost Plan, or Program of All-Inclusive Care for the Elderly.

In the matched cohort, the 6-month cumulative incidence of PC was 11.0% and 10.4% among MA and TM beneficiaries, respectively (Figure 1B). In adjusted analyses, there was no overall difference in PC receipt between MA and TM (AHR, 1.05; 95% CI, 0.98-1.12; *P* = .19). The AHR was attenuated for HMO plans (AHR, 1.16; 95% CI, 1.08-1.24; *P* < .001) (Figure 2 and eTable 6 in Supplement 1).

### PC, Systemic Therapy, and Hospice

Among the decedent subcohort with Part D coverage (n = 76 845), we similarly observed differences in PC receipt without matching (AHR, 1.34; 95% CI, 1.28-1.39; *P* < .001) and no differences after matching (AHR, 0.98; 95% CI, 0.91-1.07; *P* = .70) (eFigure 3 in Supplement 1). Systemic therapy receipt from diagnosis to death was lower in MA than in TM (66.3% vs 71.6%), especially in HMO (64.6%) and POS (64.7%) plans (Figure 3). Among all decedents (n = 100 481), hospice enrollment was higher in MA than in TM (74.1% vs 71.9%) (Figure 3). Prior to matching, MA beneficiaries were less likely than TM beneficiaries to receive systemic therapy (adjusted probability difference, −5.38 percentage points [pp]; 95% CI, −6.02 to −4.74 pp; *P* < .001) and more likely to enroll in hospice (adjusted probability difference, 3.42 pp; 95% CI, 2.82-4.03 pp; *P* < .001). Differences in systemic therapy receipt were largest for HMO (−7.32 pp; 95% CI, −8.09 to −6.54 pp; *P* < .001) and POS (−6.78 pp; 95% CI, −9.44 to −4.11 pp; *P* < .001) plans compared with TM. Differences in hospice enrollment were largest for HMO (4.09 pp; 95% CI, 3.40-4.78 pp; *P* < .001) and local PPO (2.94 pp; 95% CI, 1.94-3.94 pp; *P* < .001) plans compared with TM (Figure 4 and eTables 7 and 8 in Supplement 1). After matching, differences in adjusted probability of systemic therapy for MA vs TM persisted (−5.70 pp; 95% CI, −6.89 to −4.52 pp; *P* < .001). Differences in hospice enrollment for MA vs TM were smaller (1.68 pp; 95% CI, 0.56-2.80 pp; *P* = .003) (Figure 4 and eTable 7 in Supplement 1). When stratified by plan type, differences in adjusted probability of hospice enrollment were also attenuated for HMO (1.49 pp; 95% CI, 0.15-2.84 pp; *P* = .03) and local PPO (2.23 pp; 95% CI, 0.76-3.69 pp; *P* = .003) plans compared with TM (Figure 4 eTable 8 in Supplement 1).

**Figure 3.**
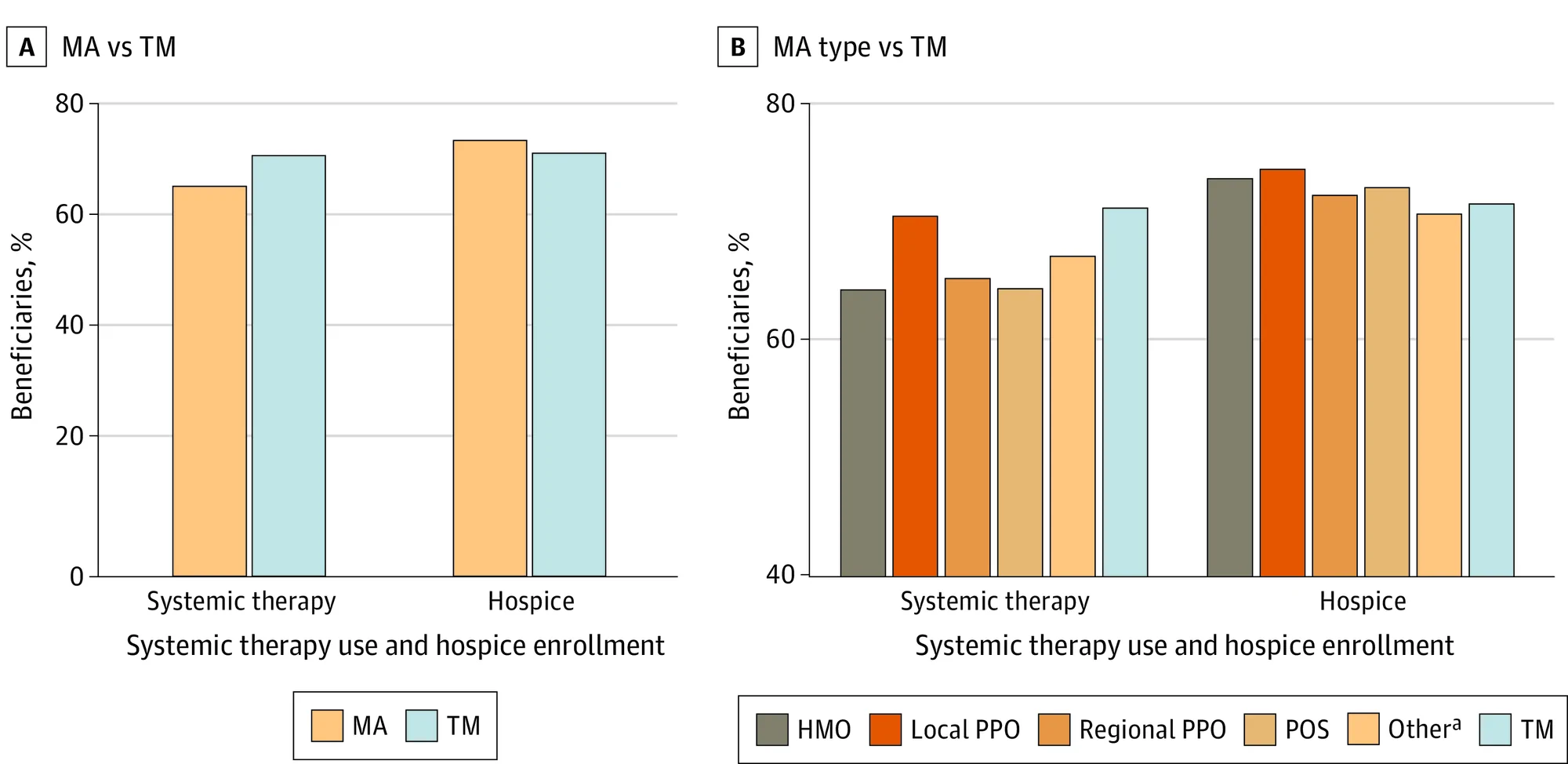
Bar Graphs Showing Proportions of Decedents With Systemic Therapy Receipt and Hospice Enrollment HMO indicates health maintenance organization; MA, Medicare Advantage; POS, point of service; PPO, preferred provider organization; TM, traditional Medicare. ^a^Other plan types included medical savings account, private fee-for-service plan, Section 1876 Cost Plan, or Program of All-Inclusive Care for the Elderly.

**Figure 4.**
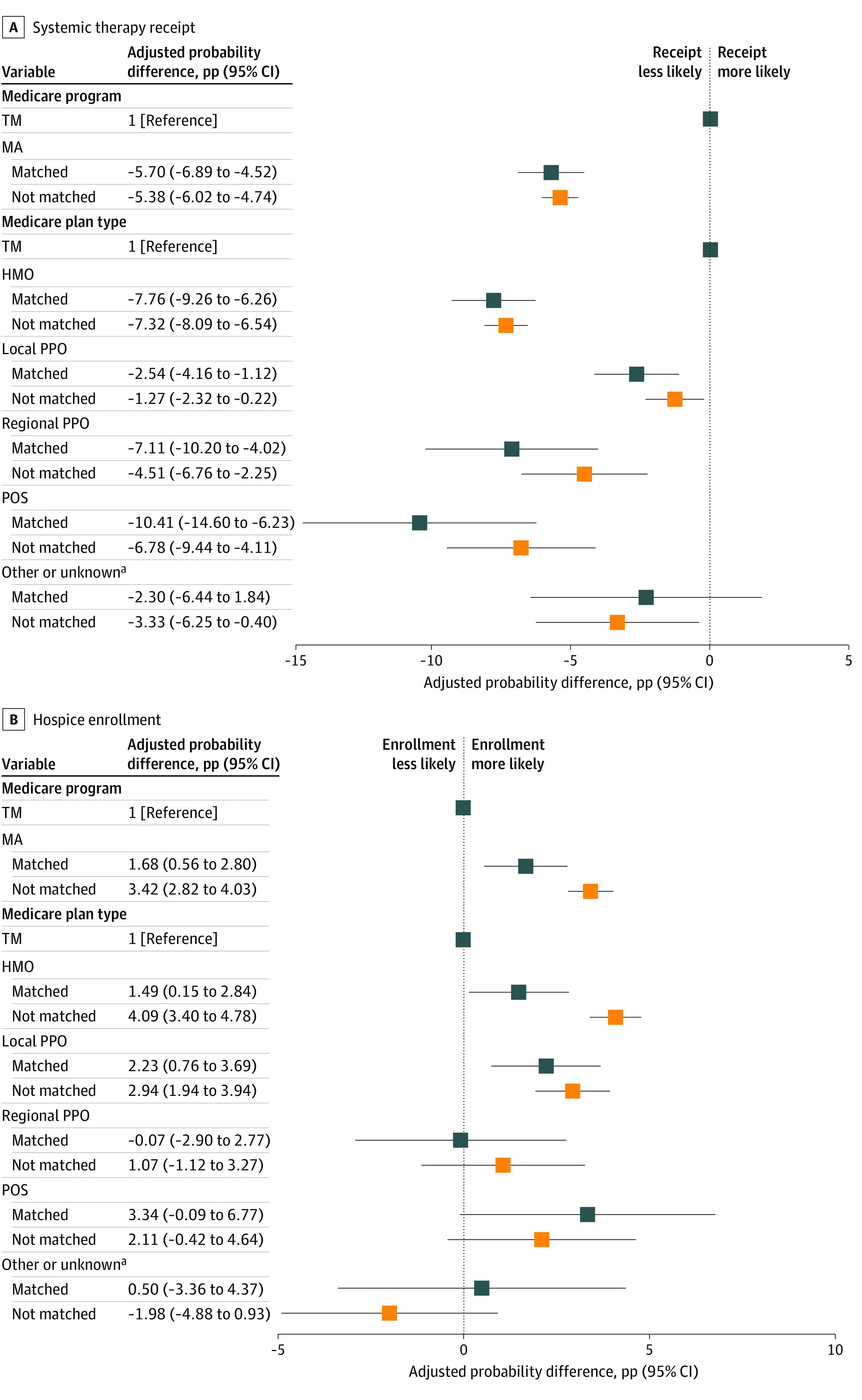
Dot-and-Whisker Plots of Adjusted Differences in Systemic Therapy Receipt and Hospice Enrollment Among Decedents Before and After Matching Based on Treating Oncologists Exact 1:1 matching of Medicare Advantage (MA) beneficiaries under each contract to traditional Medicare (TM) beneficiaries treated by the same oncologists was done. The sample size without matching was 135 402 and with matching was 46 066. The regression model adjusted for patient age, sex, race and ethnicity, metropolitan residence, marital status, dual eligibility, Yost Index quintile, cancer site, year of diagnosis, and National Cancer Institute Comorbidity Index. SEs were clustered to the beneficiary level in the matched cohort because some TM beneficiaries were matched to multiple MA beneficiaries. Whiskers represent 95% CIs. HMO indicates health maintenance organization; POS, point of service; PPO, preferred provider organization. ^a^Other plan types included medical savings account, private fee-for-service plan, Section 1876 Cost Plan, or Program of All-Inclusive Care for the Elderly.

### Sensitivity Analyses

Sensitivity analyses among beneficiaries who survived 6 or more months and those who were diagnosed and died outside the COVID-19 disruption period showed similarly higher PC utilization among MA beneficiaries than among TM beneficiaries (eFigures 4 and 5 in Supplement 1), with attenuated differences or no differences after matching (eTables 9 and 10 in Supplement 1). Stratification by cancer sites showed higher PC use among MA beneficiaries than among TM beneficiaries across all cancer sites, with attenuated differences or no differences after matching (eFigure 6 in Supplement 1). Similar patterns were found for systemic therapy and hospice enrollment across cancer sites (eFigure 7 in Supplement 1). Restricting MA beneficiaries to contracts with high data completeness and no 100% missing individual NPIs showed consistent, although smaller, differences between MA and TM (AHR, 1.12; 95% CI, 1.07-1.17; *P* < .001) (eTable 11 in Supplement 1). This suggests that data completeness is unlikely to explain the observed differences because more complete data capture would be expected to increase rather than decrease the AHR. Results excluding the NCI Comorbidity Index from regressions were also similar (eTable 12 in Supplement 1). The alternative shared-physician design showed consistent results that differences in PC receipt between MA and TM were attenuated after controlling for oncologist fixed effects (HR, 1.08; 95% CI, 1.03-1.13) compared with models without oncologist fixed effects (HR, 1.38; 95% CI, 1.33-1.43). Patterns for systemic therapy and hospice enrollment also remained similar to those in the primary analysis (eTables 13 and 14 in Supplement 1).

## Discussion

In this population-based study of Medicare beneficiaries diagnosed with common distant-stage cancers from 2016 to 2021, PC utilization within 6 months of diagnosis was 39% higher in MA (particularly in HMO plans) than in TM, a magnitude comparable to socioeconomic disparities in PC use (ie, the Yost Index) (eTable 5 in Supplement 1). This difference was largely eliminated after matching by treating oncologists. Conversely, systemic therapy receipt remained substantially lower in MA, and higher hospice enrollment differences were attenuated but persisted after matching by oncologists.

These findings suggest that specialty PC is particularly sensitive to the organizational structure of the care delivery system. Access to PC often depends on whether PC clinicians are available within the same organization where oncology care is delivered,^6^ and PC capacity is unevenly distributed and frequently concentrated in academic centers and larger organizations.^43,44^ Consistent with this, the higher PC utilization observed among MA beneficiaries was concentrated in HMO plans, many of which, including the Kaiser Foundation Health Plan, feature more selective physician contracting and integrated delivery systems with embedded PC programs that may facilitate PC referrals.^45,46^ As anticipated, unmatched MA beneficiaries were disproportionately enrolled in integrated HMO plans, which reflect closed networks in which oncologists rarely treat TM beneficiaries.^47^ These findings highlight that organizational structure may independently influence access to PC in addition to network inclusion. As vertical integration between insurers and health care organizations continues to expand,^48^ future analyses that jointly consider delivery system structure and MA network may clarify which features are associated with PC access and downstream outcomes. Higher PC utilization measured in claims, however, cannot be interpreted as more appropriate or goal-concordant referrals and also warrants future research based on patient-reported outcomes.

The persistently lower systemic therapy receipt among MA beneficiaries after oncologist matching, which removed shared oncologist confounding, suggests that utilization management (eg, prior authorization and formulary rules)^49,50^ may affect initiation or continuation of therapy even within the same oncology practice. Unobserved patient characteristics, such as performance status and disease burden, may also contribute to differences in systemic therapy receipt. The partial persistence of hospice enrollment differences after matching suggests a more multifactorial pathway shaped by patient preferences and caregiver capacity not fully driven by treating oncologists.^51^ Higher hospice enrollment was only observed in HMO and local PPO plans, not in other MA plans (compared with TM), with attenuation in such differences in the matched cohort, which was potentially attributable to the tighter care management and more structured referral pathways that may facilitate earlier hospice transitions by HMO and local PPO plans. This combination of higher specialty PC use alongside lower systemic therapy receipt and higher hospice enrollment also raises an important alternative interpretation. In particular, the financial incentives created by hospice carve-out benefits may have facilitated earlier hospice election with systemic therapy consequently forgone. It is also possible that the observed differences reflect divergent patient preferences or clinical trajectories that are not fully captured in claims. Future research to assess other patient-centered outcomes, such as alignment of goal-concordant care with patients’ symptom burden, prognosis, and preferences, is critically needed.

Our findings have important policy implications as MA enrollment continues expanding. Although PC utilization was higher among individuals with MA HMO plans compared with TM, the absolute utilization remained low (approximately 10% by 6 months). Because specialty PC capacity is concentrated in a limited number of organizations, this already underused service is particularly vulnerable to structural barriers: plans that exclude organizations with established PC programs may substantially constrain access for their enrollees, even as average utilization under MA appears to be favorable. Current monitoring of MA network adequacy covers 29 specialties, including oncology,^52^ but does not explicitly consider PC specialist availability, which is a gap that our findings suggest warrants attention. Moreover, the attenuated PC differences combined with persistent systemic therapy differences after matching suggests that evaluations of MA performance in oncology should consider network design, utilization management, and other plan features with potentially divergent effects on different aspects of cancer care.

### Limitations

Our study has limitations. First, given the observational nature, residual confounding was possible because claim-based measures do not capture symptom severity, functional status, patient preferences, or informal caregiver support, which influence PC referrals. Second, our systemic therapy and hospice measures captured utilization but not appropriateness or goal concordance. Lower systemic therapy receipt and differences in hospice enrollment may reflect unmeasured plan-level utilization management, care coordination, patient preferences, or differences in access and affordability. Third, recovering missing NPIs using the MD-PPAS file may have excluded predominantly MA-serving physicians, as MD-PPAS is derived from fee-for-service claims.^53^ The encryption of individual NPIs in SEER-Medicare data prevents linkage to external sources. However, these predominantly MA-serving physicians likely fall outside the shared-physician population that our matched analysis targeted. Fourth, we excluded beneficiaries who switched plans to ensure that exposure to a physician network and other plan-level structures remained consistent. Beneficiaries who switch plans may represent a distinct group with unmet needs and warrant future research. Fifth, the study cohort included beneficiaries in SEER regions and with selected cancers. Results may not generalize to other populations or to more recent years as MA enrollment and delivery models continue to evolve.

## Conclusions

In this retrospective cohort study of Medicare beneficiaries diagnosed with the most common distant-stage cancers, specialty PC use was higher among MA beneficiaries, particularly beneficiaries in HMO plans, than among TM beneficiaries. This difference largely disappeared after matching beneficiaries by treating oncologists, suggesting that oncologist networks may be key mechanisms affecting specialty PC receipt. In contrast, differences in systemic therapy receipt and hospice enrollment remained even after matching by treating oncologists, suggesting that plan-level policies beyond network composition may contribute to differences in treatment intensity and end-of-life transitions. As MA enrollment continues to grow, efforts to evaluate and refine MA plan design should consider network adequacy alongside other plan features that shape different components of cancer care delivery.

## Notes

Supplement 1. **eTable 1.** Cancer Identification Codes **eFigure 1.** CONSORT Chart for Creation of Patient Cohort **eTable 2.** Percentage of Claims With Missing Individual NPI by File Type **eMethods 1.** Recovering Missing Individual NPI Using Medicare Data on Provider Practice and Specialty File **eFigure 2.** Missingness of Provider Identifiers on Medicare Advantage Plan Level **eTable 3.** Systemic Therapy Identification Codes **eMethods 2.** Alternative Shared-Physician Design **eTable 4.** Sample Characteristics Among Traditional Medicare and Medicare Advantage Beneficiaries Who Were Matched and Not Matched Based on Treating Oncologists **eTable 5.** Cox Proportional Hazards Regression Model for Palliative Care Comparison Between Medicare Advantage and Traditional Medicare Before and After Matching Based on Treating Oncologists **eTable 6.** Cox Proportional Hazards Regression Model for Palliative Care Comparison Between Medicare Advantage Plan Types and Traditional Medicare Before and After Matching Based on Treating Oncologists **eFigure 3.** Cumulative Incidence of Palliative Care Up to 6 Months Before and After Matching Based on Treating Oncologists Among Subsample With Part D Coverage and Died by 2021 **eTable 7.** Logistic Regression for Systemic Therapy Receipt and Hospice Enrollment Comparison Between Medicare Advantage and Traditional Medicare Before and After Matching Based on Treating Oncologists **eTable 8.** Logistic Regression for Systemic Therapy Receipt and Hospice Enrollment Comparison Between Medicare Advantage Plan Types and Traditional Medicare Before and After Matching Based on Treating Oncologists **eFigure 4.** Cumulative Incidence of Palliative Care up to 6 Months Before and After Matching Based on Treating Oncologists Among Beneficiaries With 6 or More Months of Survival **eFigure 5.** Cumulative Incidence of Palliative Care up to 6 Months Before and After Matching Based on Treating Oncologists Among Beneficiaries Who Were Diagnosed and Died Outside of COVID-19 Disruption Period **eTable 9.** Cox Proportional Hazards Regression Model for Palliative Care Receipt up to 6 Months Before and After Matching Based on Treating Oncologists Among Beneficiaries With ≥6 Months’ Survival **eTable 10.** Cox Proportional Hazards Regression Model for Palliative Care Receipt up to 6 Months Before and After Matching Based on Treating Oncologists Among Beneficiaries Diagnosed and Died Outside COVID-19 Disruption Period **eFigure 6.** Cox Proportional Hazards Regression Model Comparing Palliative Care Receipt up to 6 Months Between Medicare Advantage and Traditional Medicare Before and After Matching Based on Treating Oncologists by Cancer Site **eFigure 7.** Systemic Therapy Receipt and Hospice Enrollment Comparison Between Medicare Advantage and Traditional Medicare Before and After Matching Based on Treating Oncologists by Cancer Site **eTable 11.** Cox Proportional Hazards Regression Model for Palliative Care Comparison Between Medicare Advantage and Traditional Medicare Among Plans With High Data Completeness and Without 100% Missing Provider IDs in Outpatient and Carrier Files **eTable 12.** Cox Proportional Hazards Regression Model for Palliative Care Comparison Between Medicare Advantage and Traditional Medicare With and Without Controlling for NCI Comorbidity Index **eTable 13.** Sample Characteristics With and Without Propensity Score Weighting for Beneficiary-Level Characteristics **eTable 14.** Palliative Care, Systemic Therapy, and Hospice Enrollment Comparison Between Medicare Advantage and Traditional Medicare With and Without Controlling for Treating Oncologist Fixed Effects Using Propensity Score Weighted Sample

Supplement 2.
Data Sharing Statement
