# Supplementary material for: Palliative Care for Advanced Cancer in Medicare Advantage and Physician Networks
Source: JAMA Netw Open. 2026 Sep 30;9(9):e2636865. doi: 10.1001/jamanetworkopen.2026.36865 (PMC13628318; doi:10.1001/jamanetworkopen.2026.36865)
Supplement: Supplementary file 1 [file jamanetwopen-e2636865-s001.pdf]

Supplementary Online Content

Hu X, Jiang C, Kwon Y, et al. Palliative care for advanced cancer in Medicare Advantage and physician networks. *JAMA Netw Open*. 2026;9(9):e2636865.
doi:10.1001/jamanetworkopen.2026.36865

eTable 1. Cancer Identification Codes

eFigure 1. CONSORT Chart for Creation of Patient Cohort

eTable 2. Percentage of Claims With Missing Individual NPI by File Type

eMethods 1. Recovering Missing Individual NPI Using Medicare Data on Provider Practice and Specialty File

eFigure 2. Missingness of Provider Identifiers on Medicare Advantage Plan Level

eTable 3. Systemic Therapy Identification Codes

eMethods 2. Alternative Shared-Physician Design

eTable 4. Sample Characteristics Among Traditional Medicare and Medicare Advantage Beneficiaries Who were Matched and Not Matched Based on Treating Oncologists

eTable 5. Cox Proportional Hazards Regression Model for Palliative Care Comparison Between Medicare Advantage and Traditional Medicare Before and After Matching Based on Treating Oncologists

eTable 6. Cox Proportional Hazards Regression Model for Palliative Care Comparison Between Medicare Advantage Plan Types and Traditional Medicare Before and After Matching Based on Treating Oncologists

eFigure 3. Cumulative Incidence of Palliative Care Up to 6 Months Before and After Matching Based on Treating Oncologists Among Subsample With Part D Coverage and Died by 2021

eTable 7. Logistic Regression for Systemic Therapy Receipt and Hospice Enrollment Comparison Between Medicare Advantage and Traditional Medicare Before and After Matching Based on Treating Oncologists

eTable 8. Logistic Regression for Systemic Therapy Receipt and Hospice Enrollment Comparison Between Medicare Advantage Plan Types and Traditional Medicare Before and After Matching Based on Treating Oncologists

eFigure 4. Cumulative Incidence of Palliative Care up to 6 Months Before and After Matching Based on Treating Oncologists Among Beneficiaries With ≥6 Months' Survival

eFigure 5. Cumulative Incidence of Palliative Care up to 6 Months Before and After Matching Based on Treating Oncologists Among Beneficiaries Diagnosed and Died Outside COVID-19 Disruption Period

© 2026 Hu X et al. *JAMA Network Open*.

eTable 9. Cox Proportional Hazards Regression Model for Palliative Care Receipt up to 6 Months Before and After Matching Based on Treating Oncologists Among Beneficiaries With ≥6 Months' Survival

eTable 10. Cox Proportional Hazards Regression Model for Palliative Care Receipt up to 6 Months Before and After Matching Based on Treating Oncologists Among Beneficiaries Diagnosed and Died Outside COVID-19 Disruption Period

eFigure 6. Cox Proportional Hazards Regression Model Comparing Palliative Care Receipt up to 6 Months Between Medicare Advantage and Traditional Medicare Before and After Matching Based on Treating Oncologists by Cancer Site

eFigure 7. Systemic Therapy Receipt and Hospice Enrollment Comparison Between Medicare Advantage and Traditional Medicare Before and After Matching Based on Treating Oncologists by Cancer Site

eTable 11. Cox Proportional Hazards Regression Model for Palliative Care Comparison Between Medicare Advantage and Traditional Medicare Among Plans with High Data Completeness and Without 100% Missing Provider IDs in Outpatient and Carrier Files

eTable 12. Cox Proportional Hazards Regression Model for Palliative Care Comparison Between Medicare Advantage and Traditional Medicare With and Without Controlling for NCI Comorbidity Index

eTable 13. Sample Characteristics With and Without Propensity Score Weighting for Beneficiary Level Characteristics

eTable 14. Palliative Care, Systemic Therapy, and Hospice Enrollment Comparison Between Medicare Advantage and Traditional Medicare With and Without Controlling for Treating Oncologist Fixed Effects Using Propensity Score Weighted Sample

This supplementary material has been provided by the authors to give readers additional information about their work.

© 2026 Hu X et al. *JAMA Network Open*.

eTable 1. Cancer Identification Codes

Cancer Type	Cancer Site	Histology
Female Breast	ICD-O-3: C500-C509; SEER Site-recode: 26000	8140,8141,8143,8147,8500,8501,8502,8503,8504, 8507,8508,8509,8520,8521,8522,8523,8524,8525, 8570,8571,8572,8573,8574,8575
Colorectal	ICD-O-3: C180-C189, C199, C209; SEER Site-recode: 21041-21049, 21051-21052, 21060	8140, 8210, 8211, 8220, 8221, 8260, 8261, 8262, 8263, 8470, 8480, 8481, 8490
NSCLC	ICD-O-3: C34.0-C34.3, C34.8, C34.9, C33.9; SEER Site-recode: 22030	8075,8076,8078,8140,8141,8143,8147, 8250,8251, 8252,8253,8254,8255,8260,8310,8430,8480,8481, 8560,8570,8571,8572,8573,8574
SCLC	ICD-O-3: C34.0-C34.3, C34.8, C34.9, C33.9; SEER Site-recode: 22030	8041, 8042, 8043, 8044, 8045
Pancreas	ICD-O-3: C250-C259; SEER Site-recode: 21100	8140, 8141,8143,8211,8500,8501,8503,8504,8508
Prostate	ICD-O-3: C619; SEER Site-recode: 28010	8140

* *Notes*: NSCL = Non-Small Cell Lung Cancer; SCL = Small Cell Lung Cancer.

© 2026 Hu X et al. *JAMA Network Open*.

eFigure 1. CONSORT Chart for creation of patient cohort

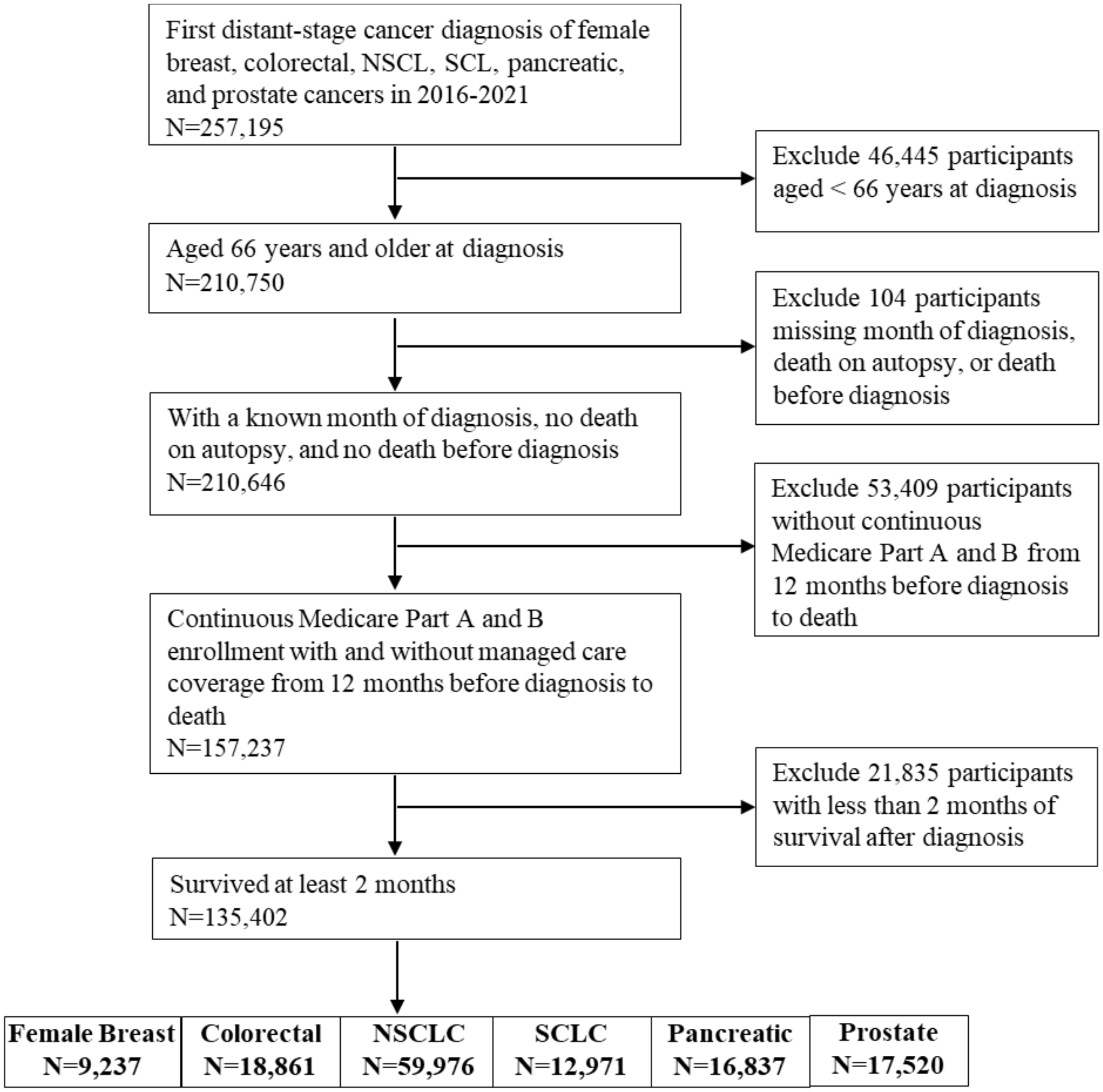

* *Notes*: NSCL = Non-Small Cell Lung Cancer; SCL = Small Cell Lung Cancer.

© 2026 Hu X et al. *JAMA Network Open*.

eTable 2. Percentage of Claims with Missing Individual NPI by File Type

File Type	Year	Total Number of Claims	% Missing Individual NPI[1]	% Missing Individual NPI after recovering[2]
Carrier Base	2016	30245316	36.5%	22.7%
Carrier Base	2017	34603832	32.9%	20.3%
Carrier Base	2018	38681563	32.8%	20.8%
Carrier Base	2019	43803264	32.8%	21.1%
Carrier Base	2020	42588508	33.3%	20.7%
Carrier Base	2021	49917452	31.1%	20.1%
Outpatient Base	2016	4279016	14.5%	
Outpatient Base	2017	4800340	14.3%	
Outpatient Base	2018	5388635	14.1%	
Outpatient Base	2019	6113449	12.4%	
Outpatient Base	2020	5902150	11.5%	
Outpatient Base	2021	7017625	10.5%	

Notes: NPI=National Provider Identifier.

[1] We used the attending physician NPIs for outpatient files, and rendering physician NPIs for carrier files.

[2] Recovering process see eMethod.

© 2026 Hu X et al. *JAMA Network Open*.

eMethods 1. Recovering Missing Individual NPI using Medicare Data on Provider Practice and Specialty File

Organization NPI data field in the Carrier Base file contain both individual NPI (Type 1) and organization NPI (Type 2). If individual NPI data field is missing, we matched the Organization NPI data field to the Medicare Data on Provider Practice and Specialty (MD-PPAS) file to identify individual NPI and recover the missing field with the matched individual NPI. Detail steps are:

1. Identify Rendering NPI data field without missing Organizational NPI field in Carrier Base file;

Patient ID	Org_npi	rndrng_npi
A001	G000001	I000001
A001	I000002	
B003	G000001	

2. Merge corresponding Organizational NPI field to MD-PPAS file to identify individual Type 1 NPI;

Patient ID	Org_npi	rndrng_npi
A001	G000001	I000001
A001	I000002	
B003	G000001	

If linked to MD-PPAS

NPI	NPI_Specialty
I000001	Medical oncology
I000002	Palliative care
I000003	Surgical oncology

3. Recover the missing Rendering NPI data field with the matched Organizational NPI field:

Patient ID	Org_npi	rndrng_npi
A001	G000001	I000001
A001	I000002	I000002
B003	G000001	

Recover

NPI	NPI_Specialty
I000001	Medical oncology
I000002	Palliative care
I000003	Surgical oncology

© 2026 Hu X et al. *JAMA Network Open*.

eFigure 2. Missingness of Provider Identifiers on Medicare Advantage Plan Level

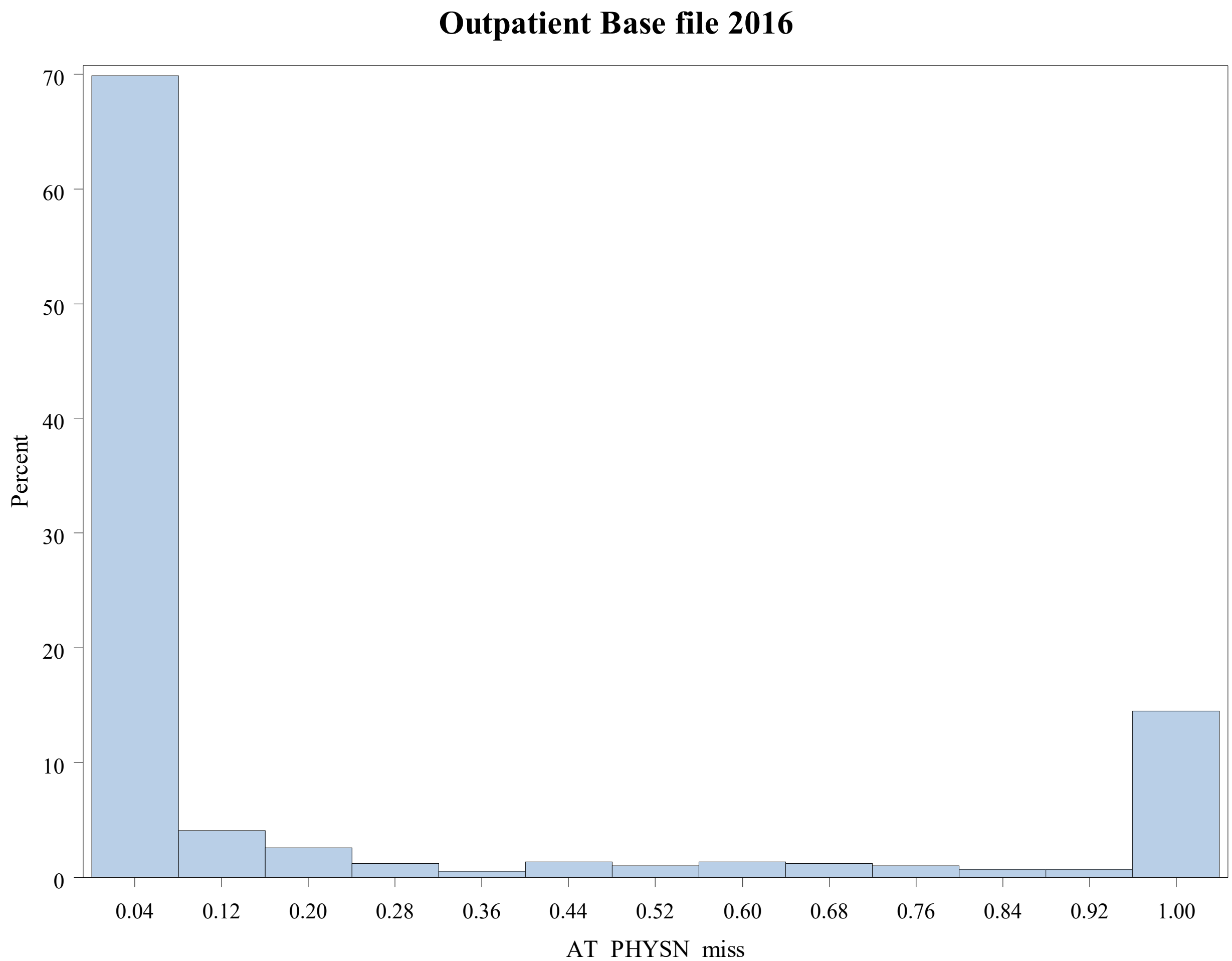

**Notes:* example using outpatient base file in 2016. Attending physician provider identifier is the most complete field and is used to examine missingness on the Medicare Advantage plan (Contract ID) level. Similar check was conducted for other years and carrier base file.

© 2026 Hu X et al. *JAMA Network Open*.

eTable 3. Systemic Therapy Identification Codes

Therapy Type	Code System	Code
Chemotherapy Infusion	ICD-9 Diagnosis Codes	V58.11, V58.12
	ICD-10 Diagnosis Codes	Z51.11, Z51.12
	ICD-9 Procedure Codes	99.25
	ICD-10 Procedure Codes	3E03305, 3E04305, XW03351, XW033B3, XW033C3, XW04351, XW043B3, XW043C3
	Revenue Center Code	0331, 0332, 0335
	HCPCS	36260, 36640, 96400, 96401, 96402, 96405, 96406, 96408, 96409, 96410, 96411, 96412, 96413, 96415, 96416, 96417, 96420, 96422, 96423, 96425, 96440, 96446, 96450, 96521, 96522, 96523, 96542, 96549

Therapy Type	Generic Name	Brand Name	HCPCS
Chemotherapy	Cabazitaxel	Jevtana	C9276, J9043, J9064
	Capecitabine	Xeloda	J8520, J8521
	Carboplatin	Carboplatin	J9045
	Cisplatin	Platinol	C9418, J9060, J9062
	Cyclophosphamide	Cyclophosphamide	J9091, J9070, J9092, J9080, J9090, C9420, C9421, J9093, J9094, J9095, J9096, J9097, J8530, C9087, J9071, J9076, J9073, J9072, J9074
	Dacarbazine	Dacarbazine	
	Docetaxel	Docefez, Taxotere	J9170, J9171, J9174, J9172
	Doxorubicin	Adriamycin, Doxil	J9000, C9415, J9002, Q2048, Q2049, Q2050, J9001
	Epirubicin	Ellence	J9178, J9180
	Eribulin	Halaven	C9280, J9179
	Estramustine	Emcyt, Estracyt	
	Etoposide	Toposar, Etopophos	C9414, C9425, J8560, J9181, J9182
	Floxuridine	Floxuridine	
	Fluorouracil	Adrucil, Carac, Efudex, Tolak, Fluoroplex	J9190
	Gemcitabine	Gemzar, Infugem	J9201, J9198, J9196
	Trifluridine and Tipiracil	Lonsurf	
	Irinotecan	Camptosar	C9474, J9206
	Ixabepilone	Ixempra	C9240, J9207
	Lurbinectedin	Zepzelca	J9223
	Mechlorethamine	Mustargen	J9230
	Methotrexate	Otrexup, Xatmep, Rasuvo, Trexall	J8610, J9250, J9260, J9255, J8611, J8612
	Mitoxantrone	Novantrone	J9293
	Oxaliplatin	Eloxatin	C9205, J9263
	Nab-paclitaxel	Abraxane	J9264
	Paclitaxel	Taxol	C9127, C9431, J9265, J9267, J9259, J9258

© 2026 Hu X et al. *JAMA Network Open*.

	Pemetrexed	Alimta, Pemfexy	C9213, J9304, J9305, J9296, J9322, J9294, J9297, J9314, J9292, J9324, J9323
	Thiotepa	Tepadina, Tepylute	C9433, J9340, J9341
	Topotecan	Hycamtin	J8705, J9350, J9351
	Trilaciclib	Cosela	C9078, J1448
	Vinblastine	Velban	J9360
	Vincristine	Oncovin, Vincasar PES, and Vincrex	J9370, J9371, J9375, J9380
	Vinorelbine	Navelbine	C9440, J9390
Hormone therapy	Abiraterone acetate	Zytiga, Yonsa	
	Anastrozole	Arimidex	S0170
	apalutamide	Erleada	
	Bicalutamide	Casodex	
	darolutamide	Nubeqa	
	Degarelix	Firmagon	J9155
	Enzalutamide	Xtandi	
	Exemestane	Aromasin	S0156
	Flutamide	Eulexin	
	Fulvestrant	Faslodex	J9395, J9394, J9393
	Goserelin	Zoladex	J9202
	Ketoconazole	Nizoral	
	Letrozole	Femara	
	Leuprolide	Lupron, Eligard, Camcevi	C9430, J1950, J9217, J9218, J9219, J1952
	Leuprolide acetate for depot suspension (lutrate depot)	Lutrate Depot	J1954
	Megestrol Acetate	Megace	
	Nilutamide	Nilandron	
	Relugolix	Orgovyx	
	Tamoxifen	Soltamox	S0187
	Toremifene	Fareston	
	Triptorelin	Trelstar	J3315
Immunotherapy	Atezolizumab	Tecentriq	C9483, J9022, J9024
	Cemiplimab-rwlc	Libtayo	J9113
	Cosibelimab	Unloxcyt	
	Datopotamab Deruxtecan	Datroway	J9011, C9174
	dostarlimab	Jemperli	C9082, J9272
	Durvalumab	Imfinzi	C9492, J9173
	Ipilimumab	Yervoy	C9284, J9228
	Nivolumab	Opdivo	J9299, C9453
	Nivolumab and hyaluronidase-nvhy	Opdivo Qvantig	J9289
	Pembrolizumab	Keytruda	J9271, C9027
	Sipuleucel-T	Provenge	C9273, Q2043
	Tarlatamab-dlle	Imdelltra	J9026, C9170
	Telisotuzumab vedotin-tllv	Emrelis	C9306
	Tislelizumab-jsgr	Tevimbra	J9329
	Tremelimumab-actl	Imjudo	C9147, J9347
Other	lutetium Lu 177 vipivotide tetraxetan	Pluvicto	

© 2026 Hu X et al. *JAMA Network Open*.

	Necitumumab	Portrazza	C9475, J9295
	Radium Ra 223 dichloride	Xofigo	
Targeted therapy	Abemaciclib	Verzenio	
	Adagrasib	Krazati	
	Ado-Trastuzumab Emtansine	Kadcyla	C9131, J9354
	Afatinib	Gilotrif	
	Alectinib	Alecensa	
	Alpelisib	Piqray	
	Amivantamab	Rybrevant	J9061, C9083
	Bevacizumab	Avastin, Mvasi, Zirabev	Q2024, C9257, C9214, J9035, S0116, Q5118, Q5107
	Bevacizumab-adcd	Vegzelma	Q5129
	Bevacizumab-maly	Alymsys	Q5126, C9142
	Brigatinib	Alunbrig	
	capivasertib	Truqap	
	Capmatinib	Tabrecta	
	Ceritinib	Zykadia	
	Cetuximab	Erbitux	C9215, J9055
	Crizotinib	Xalkori	
	Dabrafenib	Tafinlar	
	Elacestrant	Orserdu	
	Encorafenib	Braftovi	
	Ensartinib	Ensacove	
	Entrectinib	Rozlytrek	
	Erlotinib	Tarceva	
	Everolimus	Afinitor	J7527, J8561
	Fam-Trastuzumab Deruxtecan-nxki	Enhertu	J9358
	Fruquintinib	Fruzaqla	
	Gefitinib	IRESSA	J8565
	Inavolisib	Itovebi	
	Lapatinib	Tykerb	
	Lapatinib	Tykerb	
	Larotrectinib	Vitrakvi	
	Lazertinib	Lazcluze	
	Lorlatinib	Lorbrena	
	Margetuximab-cmkb	Margenza	J9353
	Mobocertinib	Exkivity	
	Neratinib	Nerlynx	
	Niraparib Tosylate Monohydrate and Abiraterone Acetate	Akeega	
	Olaparib	Lynparza	
	Osimertinib	Tagrisso	
	Palbociclib	Ibrance	
	Panitumumab	Vectibix	C9235, J9303
	Pertuzumab	Perjeta	C9292, J9306, J9316
	Pertuzumab, Trastuzumab, and Hyaluronidase-zzxf	Phesgo	J9316
	Pralsetinib	Gavreto	
	Ramucirumab	Cyramza	C9025, J9308

© 2026 Hu X et al. *JAMA Network Open*.

	Regorafenib	Stivarga	
	Repotrectinib	Augtyro	
	Ribociclib	Kisqali	
	Ripretinib	QINLOCK	
	Rucaparib	Rubraca	
	Sacituzumab Govitecan-hziy	Trodelvy	C9066, J9317
	Selpercatinib	Retevmo	
	sotorasib	Lumakras	
	Talazoparib	Talzenna	
	Taletrectinib	Ibtrozi	
	Tepotinib Hydrochloride	Tepmetko	
	Trametinib	Mekinist	
	Trastuzumab	Herceptin	J9355, J9356
	Trastuzumab (biosimilar)	Kanjinti, Ogivri, Ontruzant, Herzuma, Trazimera, Hercessi	Q5117, Q5114, Q5112, Q5113, Q5116, Q5146
	Tucatinib	Tukysa	
	Vemurafenib	Zelboraf	
	Zenocutuzumab-zbco	Bizengri	J9382
	Ziv-Aflibercept	Zaltrap	C9296, J9400

Notes: Healthcare Common Procedure Coding System (HCPCS) for each regimen are identified using Oncology Toolbox provided by SEER (https://seer.cancer.gov/oncologytoolbox/). Oral regimens are identified using generic name and brand name.

© 2026 Hu X et al. *JAMA Network Open*.

eMethods 2. Alternative Shared-Physician Design

Our primary analysis used exact matching on treating oncologist to isolate the contribution of provider networks to MA–TM differences. Because matching necessarily restricts the analytic sample to beneficiaries whose treating oncologists served both MA and TM populations, we implemented an alternative Shared-Physician design that retains the full analytic sample. This approach includes balancing patient composition and conditioning on treating provider as sequential steps, which allows the contribution of provider networks to be assessed while holding patient characteristics constant across the entire cohort.

Step 1. Propensity Score Weighting

We estimated the propensity for MA enrollment using logistic regression with the same individual-level sociodemographic and clinical characteristics included in our primary multivariable models: age at diagnosis, sex, race and ethnicity, marital status, metropolitan residence, neighborhood socioeconomic status (Yost quintile), dual eligibility status, cancer site, year of diagnosis, and NCI comorbidity index. Inverse probability of treatment weights targeting the average treatment effect were constructed as 1/PS for MA beneficiaries and 1/(1−PS) for TM beneficiaries.

Covariate balance was assessed using standardized mean differences (SMDs). All covariates achieved SMDs below 0.10 following weighting, indicating excellent balance between MA and TM beneficiaries (eTable 13).

Step 2. Comparison of MA-TM Differences With and Without Treating Oncologist Fixed Effects

Within the weighted sample, we estimated MA–TM differences in each study outcome using two model specifications. The first included MA enrollment as the exposure without treating oncologist adjustment, which yields the total MA–TM difference net of observed patient characteristics. The second additionally included fixed effects for treating oncologist, which restricts identification to within-oncologist variation in MA versus TM enrollment and therefore removes any component of the MA–TM difference attributable to differential sorting of beneficiaries across oncologists. The change in the MA–TM estimate between these specifications quantifies the portion of the difference attributable to provider network composition.

Time to first PC was modeled using a Cox proportional hazards model with a "STRATA" statement for treating oncologist under the SAS "PHREG" model, which allows each oncologist different baseline hazards while constraining the effect of MA enrollment and other covariates to be common across strata. Systemic therapy receipt and hospice enrollment among decedents were modeled using linear probability models with treating oncologist fixed effects. Standard errors were clustered at the treating oncologist level. Oncologists who treated only MA or only TM beneficiaries, and strata without events, do not contribute to the identification of the MA coefficient in the fixed-effects specification. Regression results with and without accounting for treating oncologist fixed effects are reported in eTable 14.

© 2026 Hu X et al. *JAMA Network Open*.

eTable 4. Sample Characteristics Among Traditional Medicare and Medicare Advantage Beneficiaries Who were Matched and Not Matched Based on Treating Oncologists

Variables	Matched			Not matched			
	MA	TM	P-value	MA	P-value (vs. MA Matched)	TM	P-value (vs. TM Matched)
**Total N**	23033 (100.0)	23033 (100.0)		20741 (100.0)		74022 (100.0)	
**Year of Diagnosis, N (Col %)**			1.0		<.001		<.001
**2016**	2936 (12.8)	2936 (12.8)		2812 (13.6)		13787 (18.6)	
**2017**	3090 (13.4)	3090 (13.4)		3056 (14.7)		13577 (18.3)	
**2018**	3623 (15.7)	3623 (15.7)		3241 (15.6)		13329 (18.0)	
**2019**	4336 (18.8)	4336 (18.8)		3846 (18.5)		12897 (17.4)	
**2020**	4493 (19.5)	4493 (19.5)		3732 (18.0)		11135 (15.0)	
**2021**	4555 (19.8)	4555 (19.8)		4054 (19.6)		9297 (12.6)	
**Age, Mean (SD)**	75.7 (6.4)	75.6 (6.5)	<.001	76.8 (6.7)	<.001	76.3 (6.8)	<.001
**Age groups at Diagnosis, N (Col %)**			0.030		<.001		<.001
**66-74**	10977 (47.7)	11240 (48.8)		8664 (41.8)		33199 (44.9)	
**75-84**	9414 (40.9)	9146 (39.7)		8776 (42.3)		29840 (40.3)	
**85+**	2642 (11.5)	2647 (11.5)		3301 (15.9)		10983 (14.8)	
**Sex, N (Col %)**			0.26		<.001		0.004
**Male**	11944 (51.9)	12064 (52.4)		11452 (55.2)		39578 (53.5)	
**Female**	11089 (48.1)	10969 (47.6)		9289 (44.8)		34444 (46.5)	
**Race and ethnicity, N (Col %)**			<.001		<.001		<.001
**Hispanic**	1995 (8.7)	1264 (5.5)		2825 (13.6)		4125 (5.6)	
**Non-Hispanic Black**	2858 (12.4)	1954 (8.5)		2929 (14.1)		5612 (7.6)	
**Non-Hispanic Other** [a]	965 (4.2)	983 (4.3)		1634 (7.9)		3143 (4.3)	
**Non-Hispanic White**	17183 (74.6)	18781 (81.5)		13308 (64.2)		61005 (82.4)	
**Unknown**	32 (0.1)	51 (0.2)		45 (0.2)		137 (0.2)	
**Metropolitan residency** [b]**, N (Col %)**			<.001		<.001		<.001
**Non-metropolitan**	2489 (10.8)	3236 (14.1)		1046 (5.0)		12389 (16.7)	
**Metropolitan**	20544 (89.2)	19797 (86.0)		19695 (95.0)		61633 (83.3)	
**Dual eligibility** [c]**, N (Col %)**			<.001		0.077		0.034
**Non-dual**	20026 (86.9)	20321 (88.2)		17914 (86.4)		64918 (87.7)	
**Dual**	3007 (13.1)	2712 (11.8)		2827 (13.6)		9104 (12.3)	
**Marital Status, N (Col %)**			0.40		<.001		<.001
**Married**	9385 (40.8)	9528 (41.4)		9401 (45.3)		29278 (39.6)	
**Not married**	7917 (34.4)	7834 (34.0)		7889 (38.0)		24976 (33.7)	

© 2026 Hu X et al. *JAMA Network Open*.

Variables	Matched			Not matched			
	MA	TM	P-value	MA	P-value (vs. MA Matched)	TM	P-value (vs. TM Matched)
**Unknown**	5731 (24.9)	5671 (24.6)		3451 (16.6)		19768 (26.7)	
**Yost Quintile [d], N (Col %)**			<.001		<.001		<.001
**Q1 (Lowest SES)**	3502 (15.2)	3004 (13.0)		3041 (14.7)		9080 (12.3)	
**Q2**	3660 (15.9)	3510 (15.2)		3046 (14.7)		10777 (14.6)	
**Q3**	4112 (17.9)	4194 (18.2)		3725 (18.0)		12158 (16.4)	
**Q4**	4794 (20.8)	4626 (20.1)		4531 (21.9)		14194 (19.2)	
**Q5 (Highest SES)**	4655 (20.2)	5434 (23.6)		5421 (26.1)		18734 (25.3)	
**Unknown**	2310 (10.0)	2265 (9.8)		977 (4.7)		9079 (12.3)	
**Cancer site, N (Col %)**			<.001		<.001		<.001
**Breast**	1391 (6.0)	1674 (7.3)		1424 (6.9)		5125 (6.9)	
**Colorectal**	3063 (13.3)	3327 (14.4)		2845 (13.7)		10385 (14.0)	
**NSCLC**	10708 (46.5)	10243 (44.5)		8859 (42.7)		32616 (44.1)	
**Pancreas**	3028 (13.2)	2889 (12.5)		2481 (12.0)		9117 (12.3)	
**Prostate**	2255 (9.8)	2416 (10.5)		3597 (17.3)		9807 (13.3)	
**SCLC**	2588 (11.2)	2484 (10.8)		1535 (7.4)		6972 (9.4)	
**NCI Comorbidity Index, Mean (SD)**	0.56 (0.59)	0.50 (0.58)	<.001	0.61 (0.61)	<.001	0.53 (0.61)	<.001
**Plan Type, N (Col %)**							
**TM**	NA	23033 (100.0)		NA		23033 (100.0)	
**HMO**	11315 (49.1)	NA		15902 (76.7)		NA	
**Local PPO**	8490 (36.9)	NA		3215 (15.5)		NA	
**Regional PPO**	1455 (6.3)	NA		534 (2.6)		NA	
**POS**	1004 (4.4)	NA		619 (3.0)		NA	
**Other/Unknown**	769 (3.3)	NA		471 (2.3)		NA	

* *Notes*: HMO = Health Maintenance Organization; MA=Medicare Advantage; NSCL = Non-Small Cell Lung; SCL = Small Cell Lung; PPO = Preferred Provider Organization; POS = Point of Service; SES = Socioeconomic Status; TM=Traditional Medicare.

[a] Other group includes American Indian/Alaska Native, Asian/Pacific Islander, and other unspecified.

[b] Based on 2013 Rural Urban Continuum Code. Codes 1-3 were classified as metro, and Codes 4-9 were classified as non-metro.

[c] With state buy-in (Medicaid) from cancer diagnosis to the end of follow-up.

[d] Yost index is a composite SES scores for census tracts based on Median household income, Median house value, Median rent, Percent below 150% of federal poverty line, Education Index, Percent working class, and Percent unemployed. Lower quintiles represent the lower SES groups.

© 2026 Hu X et al. *JAMA Network Open*.

eTable 5. Cox Proportional Hazards Regression Model for Palliative Care Comparison Between Medicare Advantage and Traditional Medicare Before and After Matching Based on Treating Oncologists

Variables	Without Matching		Matched	
	HR (95%CI)	P-value	HR (95%CI)	P-value
**Total N**	**135,402**		**46,066**	
**Medicare Plan Type**				
**TM**	Ref		Ref	
**MA**	1.39 (1.35, 1.44)	<.001	1.05 (0.98, 1.12)	0.19
**Year of Diagnosis**				
**2016**	Ref		Ref	
**2017**	1.22 (1.14, 1.30)	<.001	1.17 (1.02, 1.35)	0.026
**2018**	1.48 (1.39, 1.57)	<.001	1.41 (1.23, 1.61)	<.001
**2019**	1.61 (1.52, 1.71)	<.001	1.59 (1.40, 1.81)	<.001
**2020**	1.60 (1.50, 1.70)	<.001	1.61 (1.41, 1.83)	<.001
**2021**	1.80 (1.69, 1.91)	<.001	1.74 (1.53, 1.97)	<.001
**Age groups**				
**66-74**	Ref		Ref	
**75-84**	0.94 (0.91, 0.97)	0.001	0.89 (0.83, 0.95)	0.001
**85+**	0.95 (0.90, 1.00)	0.061	0.91 (0.81, 1.02)	0.091
**Sex**				
**Male**	Ref		Ref	
**Female**	1.07 (1.03, 1.11)	<.001	1.00 (0.93, 1.07)	0.98
**Race and ethnicity**				
**Non-Hispanic White**	Ref		Ref	
**Hispanic**	1.15 (1.08, 1.23)	<.001	1.12 (0.99, 1.26)	0.075
**Non-Hispanic Black**	0.99 (0.93, 1.06)	0.81	1.00 (0.89, 1.12)	0.98
**Non-Hispanic Other**[a]	1.10 (1.03, 1.19)	0.008	0.94 (0.79, 1.11)	0.45
**Unknown**	0.84 (0.55, 1.29)	0.43	0.66 (0.23, 1.85)	0.43
**Metropolitan residency** [b]				
**Non-metropolitan**	Ref		Ref	
**Metropolitan**	1.10 (1.04, 1.17)	0.001	1.10 (0.98, 1.23)	0.11
**Dual eligibility** [c]				
**Non-dual**	Ref		Ref	
**Dual**	1.04 (0.99, 1.10)	0.14	1.14 (1.03, 1.27)	0.009
**Marital Status**				
**Married**	Ref		Ref	
**Not married**	1.06 (1.02, 1.11)	0.002	1.10 (1.02, 1.19)	0.012
**Unknown**	0.78 (0.74, 0.82)	<.001	0.77 (0.70, 0.86)	<.001
**Yost Quintile** [d]				
**Q1 (Lowest SES)**	Ref		Ref	
**Q2**	1.13 (1.06, 1.21)	<.001	0.96 (0.85, 1.10)	0.57
**Q3**	1.23 (1.15, 1.31)	<.001	1.16 (1.02, 1.31)	0.020
**Q4**	1.33 (1.24, 1.42)	<.001	1.17 (1.03, 1.32)	0.013
**Q5 (Highest SES)**	1.60 (1.50, 1.70)	<.001	1.42 (1.26, 1.60)	<.001
**Unknown**	1.33 (1.22, 1.45)	<.001	1.22 (1.04, 1.43)	0.013
**Cancer site**				
**Breast**	Ref		Ref	
**Colorectal**	0.84 (0.76, 0.91)	<.001	0.80 (0.66, 0.97)	0.022
**NSCLC**	1.35 (1.28, 1.43)	<.001	1.48 (1.32, 1.66)	<.001

© 2026 Hu X et al. *JAMA Network Open*.

Variables	Without Matching		Matched	
	HR (95%CI)	P-value	HR (95%CI)	P-value
**Pancreas**	1.86 (1.75, 1.97)	<.001	2.03 (1.79, 2.30)	<.001
**Prostate**	0.58 (0.54, 0.63)	<.001	0.62 (0.51, 0.74)	<.001
**SCLC**	1.35 (1.26, 1.45)	<.001	1.45 (1.26, 1.66)	<.001
**NCI Comorbidity Index**	0.98 (0.95, 1.00)	0.081	0.97 (0.92, 1.03)	0.30

* *Notes*: MA=Medicare Advantage; NSCL = Non-Small Cell Lung; SCL = Small Cell Lung; SES = Socioeconomic Status; TM=Traditional Medicare. We conducted 1:1 exact matching on treating oncologists for MA beneficiaries under each contract to TM beneficiaries treated by the same oncologists. Standard errors were clustered to the beneficiary level in the matched cohort because some TM beneficiaries we matched to multiple MA beneficiaries.

[a] Other group includes American Indian/Alaska Native, Asian/Pacific Islander, and other unspecified.

[b] Based on 2013 Rural Urban Continuum Code. Codes 1-3 were classified as metropolitan, and Codes 4-9 were classified as non-metropolitan.

[c] With state buy-in (Medicaid) from cancer diagnosis to the end of follow-up.

[d] Yost index is a composite SES scores for census tracts based on Median household income, Median house value, Median rent, Percent below 150% of federal poverty line, Education Index, Percent working class, and Percent unemployed. Lower quintiles represent the lower SES groups.

© 2026 Hu X et al. *JAMA Network Open*.

eTable 6. Cox Proportional Hazards Regression Model for Palliative Care Comparison Between Medicare Advantage Plan Types and Traditional Medicare Before and After Matching Based on Treating Oncologists

Variables	Without Matching		Matched	
	HR (95%CI)	P-value	HR (95%CI)	P-value
**Total N**	**135,402**		**46,066**	
**Medicare Plan Type**				
**TM**	Ref		Ref	
**HMO**	1.66 (1.60, 1.73)	<.001	1.16 (1.08, 1.24)	<.001
**Local PPO**	0.95 (0.89, 1.01)	0.12	0.90 (0.83, 0.98)	0.011
**Region PPO**	1.02 (0.88, 1.18)	0.84	0.97 (0.82, 1.15)	0.72
**POS**	1.15 (0.99, 1.33)	0.067	1.19 (1.00, 1.43)	0.054
**Other/Unk**	0.94 (0.77, 1.15)	0.55	0.98 (0.77, 1.25)	0.87
**Year of Diagnosis**				
**2016**	Ref		Ref	
**2017**	1.22 (1.14, 1.30)	<.001	1.17 (1.04, 1.33)	0.012
**2018**	1.47 (1.38, 1.57)	<.001	1.40 (1.25, 1.58)	<.001
**2019**	1.62 (1.52, 1.72)	<.001	1.59 (1.42, 1.78)	<.001
**2020**	1.62 (1.52, 1.72)	<.001	1.62 (1.45, 1.80)	<.001
**2021**	1.82 (1.71, 1.93)	<.001	1.74 (1.56, 1.95)	<.001
**Age groups**				
**66-74**	Ref		Ref	
**75-84**	0.94 (0.91, 0.98)	0.001	0.89 (0.84, 0.95)	<.001
**85+**	0.95 (0.90, 1.00)	0.062	0.91 (0.83, 1.00)	0.054
**Sex**				
**Male**	Ref		Ref	
**Female**	1.07 (1.03, 1.11)	<.001	1.00 (0.94, 1.06)	0.99
**Race and ethnicity**				
**Non-Hispanic White**	Ref		Ref	
**Hispanic**	1.09 (1.02, 1.16)	0.008	1.09 (0.97, 1.22)	0.14
**Non-Hispanic Black**	0.98 (0.92, 1.04)	0.50	1.00 (0.90, 1.10)	0.93
**Non-Hispanic Other** [a]	1.05 (0.97, 1.13)	0.22	0.92 (0.80, 1.05)	0.22
**Unknown**	0.81 (0.53, 1.25)	0.34	0.65 (0.27, 1.56)	0.33
**Metropolitan residency** [b]				
**Non-metropolitan**	Ref		Ref	
**Metropolitan**	1.06 (1.00, 1.12)	0.069	1.07 (0.97, 1.18)	0.19
**Dual eligibility** [c]				
**Non-dual**	Ref		Ref	
**Dual**	1.01 (0.95, 1.06)	0.81	1.11 (1.02, 1.21)	0.023
**Marital Status**				
**Married**	Ref		Ref	
**Not married**	1.06 (1.02, 1.10)	0.004	1.10 (1.03, 1.17)	0.006
**Unknown**	0.80 (0.76, 0.84)	<.001	0.78 (0.71, 0.86)	<.001
**Yost Quintile** [d]				
**Q1 (Lowest SES)**	Ref		Ref	
**Q2**	1.13 (1.06, 1.21)	<.001	0.96 (0.86, 1.08)	0.52
**Q3**	1.22 (1.14, 1.30)	<.001	1.15 (1.04, 1.28)	0.008
**Q4**	1.31 (1.23, 1.40)	<.001	1.16 (1.04, 1.29)	0.006
**Q5 (Highest SES)**	1.57 (1.48, 1.68)	<.001	1.41 (1.27, 1.57)	<.001

© 2026 Hu X et al. *JAMA Network Open*.

Variables	Without Matching		Matched	
	HR (95%CI)	P-value	HR (95%CI)	P-value
**Unknown**	1.33 (1.22, 1.45)	<.001	1.22 (1.06, 1.41)	0.006
**Cancer site**				
**Breast**	Ref		Ref	
**Colorectal**	0.83 (0.76, 0.91)	<.001	0.80 (0.68, 0.94)	0.007
**NSCLC**	1.35 (1.28, 1.43)	<.001	1.48 (1.35, 1.63)	<.001
**Pancreas**	1.86 (1.75, 1.98)	<.001	2.04 (1.83, 2.27)	<.001
**Prostate**	0.58 (0.53, 0.63)	<.001	0.61 (0.52, 0.72)	<.001
**SCLC**	1.36 (1.26, 1.46)	<.001	1.45 (1.29, 1.63)	<.001
**NCI Comorbidity Index**	0.97 (0.94, 1.00)	0.034	0.97 (0.92, 1.02)	0.23

* *Notes*: HMO = Health Maintenance Organization; NSCL = Non-Small Cell Lung; SCL = Small Cell Lung; PPO = Preferred Provider Organization; POS = Point of Service; SES = Socioeconomic Status; TM=Traditional Medicare. We conducted 1:1 exact matching on treating oncologists for MA beneficiaries under each contract to TM beneficiaries treated by the same oncologists. Standard errors were clustered to the beneficiary level in the matched cohort because some TM beneficiaries we matched to multiple MA beneficiaries.

[a] Other group includes American Indian/Alaska Native, Asian/Pacific Islander, and other unspecified.

[b] Based on 2013 Rural Urban Continuum Code. Codes 1-3 were classified as metropolitan, and Codes 4-9 were classified as non-metropolitan.

[c] With state buy-in (Medicaid) from cancer diagnosis to the end of follow-up.

[d] Yost index is a composite SES scores for census tracts based on Median household income, Median house value, Median rent, Percent below 150% of federal poverty line, Education Index, Percent working class, and Percent unemployed. Lower quintiles represent the lower SES groups.

© 2026 Hu X et al. *JAMA Network Open*.

eFigure 3. Cumulative Incidence of Palliative Care Up to 6 Months Before and After Matching Based on Treating Oncologists Among Subsample with Part D Coverage and Died by 2021

A. Without Matching

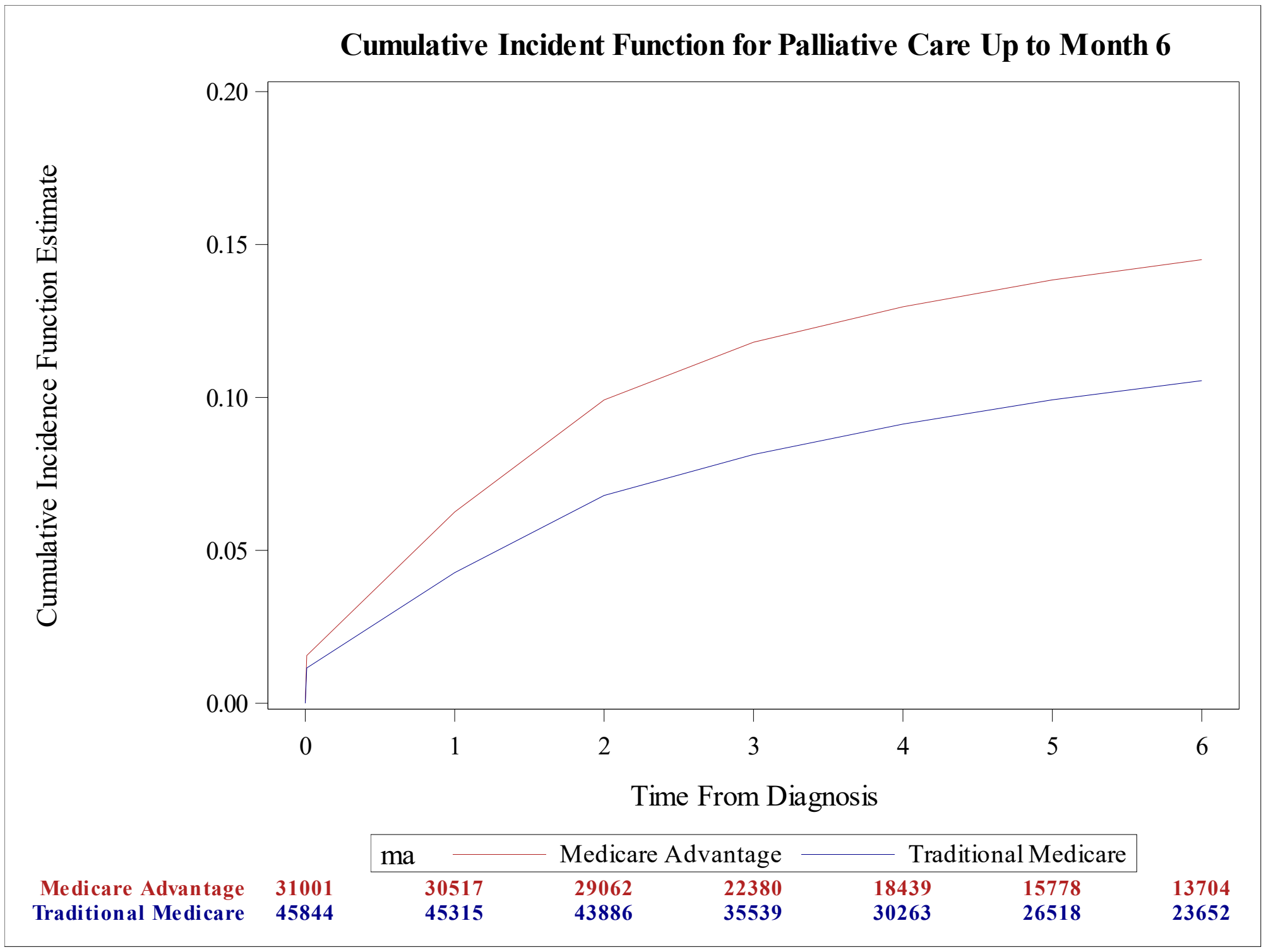

B. After Matching

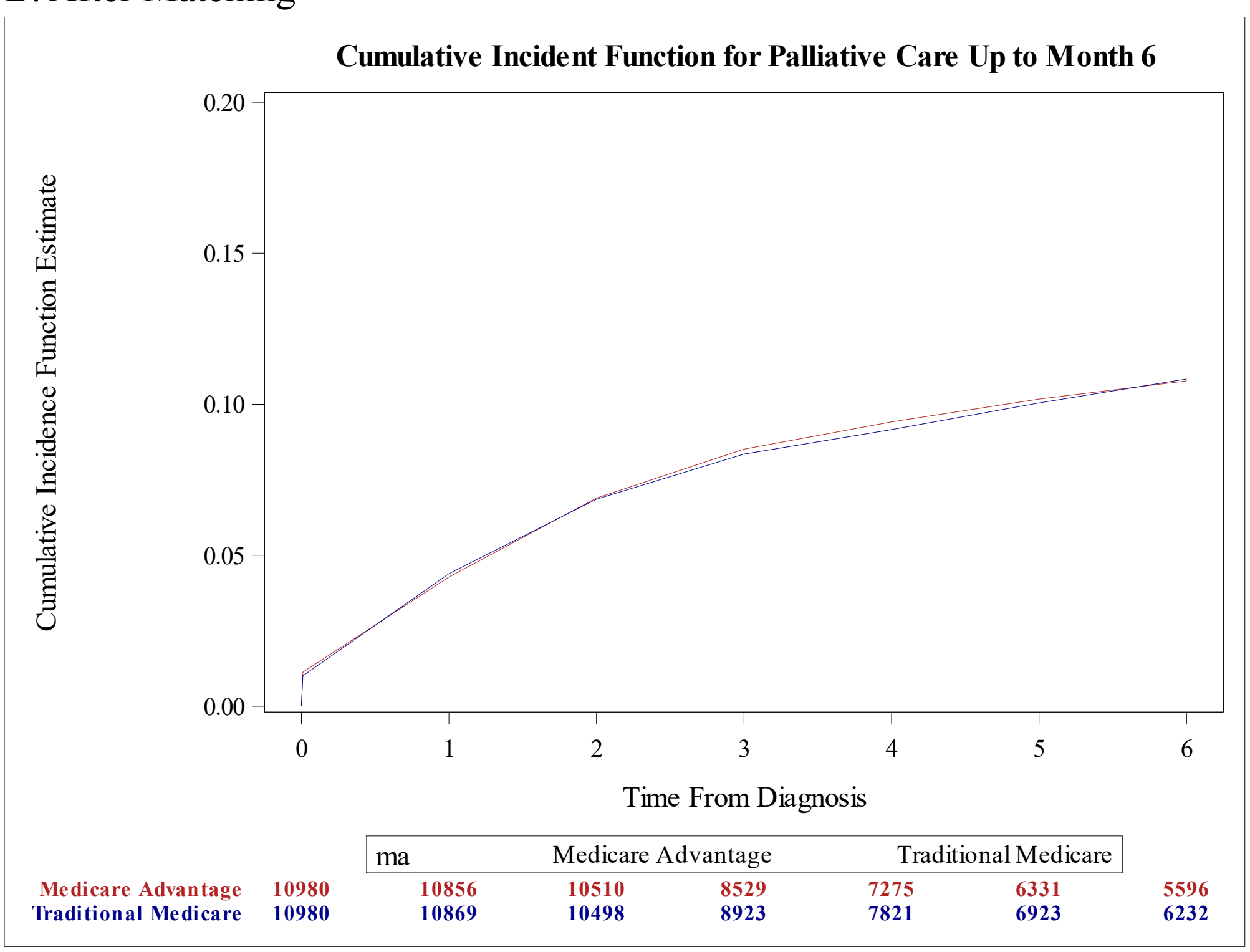

© 2026 Hu X et al. *JAMA Network Open*.

eTable 7. Logistic Regression for Systemic Therapy Receipt and Hospice Enrollment Comparison Between Medicare Advantage and Traditional Medicare Before and After Matching Based on Treating Oncologists

**Variables**	**Systemic Therapy**				**Hospice Enrollment**			
	**Without Matching**		**Matched**		**Without Matching**		**Matched**	
	**Adjusted Probability Difference (95%CI)**	**P-value**	**Adjusted Probability Difference (95%CI)**	**P-value**	**Adjusted Probability Difference (95%CI)**	**P-value**	**Adjusted Probability Difference (95%CI)**	**P-value**
**Total N**	**76,845**		**21,960**		**100,481**		**30,138**	
**Medicare Plan Type**								
**TM**	Ref		Ref		Ref		Ref	
**MA**	-5.38 (-6.02, -4.74)	<.001	-5.70 (-6.89, -4.52)	<.001	3.42 (2.82, 4.03)	<.001	1.68 (0.56, 2.80)	0.003
**Year of Diagnosis**								
**2016**	Ref		Ref		Ref		Ref	
**2017**	0.50 (-0.49, 1.50)	0.32	0.76 (-1.04, 2.55)	0.41	0.23 (-0.62, 1.08)	0.60	0.94 (-0.86, 2.74)	0.31
**2018**	1.47 (0.48, 2.46)	0.004	0.50 (-1.29, 2.28)	0.59	0.02 (-0.84, 0.88)	0.97	1.29 (-0.51, 3.09)	0.16
**2019**	0.99 (0.00, 1.98)	0.050	-0.12 (-1.90, 1.66)	0.90	-0.78 (-1.65, 0.09)	0.081	0.31 (-1.48, 2.09)	0.74
**2020**	-1.83 (-2.90, -0.76)	0.001	-1.45 (-3.96, 1.07)	0.26	-0.54 (-1.49, 0.40)	0.26	0.24 (-1.68, 2.16)	0.81
**2021**	-11.96 (-13.32, -10.60)	<.001	-10.18 (-13.26, -7.10)	<.001	-3.65 (-4.85, -2.44)	<.001	-4.12 (-6.80, -1.43)	0.003
**Age groups**								
**66-74**	Ref		Ref		Ref		Ref	
**75-84**	-8.10 (-8.77, -7.44)	<.001	-6.28 (-7.52, -5.04)	<.001	4.52 (3.91, 5.13)	<.001	4.30 (3.08, 5.52)	<.001
**85+**	-25.13 (-26.14, -24.11)	<.001	-19.45 (-21.72, -17.18)	<.001	9.55 (8.76, 10.33)	<.001	9.43 (7.72, 11.15)	<.001
**Sex**								
**Male**	Ref		Ref		Ref		Ref	
**Female**	-2.02 (-2.69, -1.36)	<.001	-2.52 (-3.63, -1.41)	<.001	5.99 (5.39, 6.60)	<.001	5.77 (4.55, 6.99)	<.001
**Race and ethnicity**								
**Non-Hispanic White**	Ref		Ref		Ref		Ref	
**Hispanic**	-1.47 (-2.64, -0.30)	0.014	-0.36 (-2.71, 1.99)	0.76	3.05 (1.92, 4.18)	<.001	4.84 (2.51, 7.17)	<.001
**Non-Hispanic Black**	-2.48 (-3.92, -1.05)	0.001	0.15 (-2.62, 2.92)	0.92	-6.21 (-7.62, -4.80)	<.001	-5.28 (-8.15, -2.41)	<.001
**Non-Hispanic Other** [a]	3.20 (1.49, 4.91)	<.001	2.26 (-1.40, 5.92)	0.23	-4.82 (-6.56, -3.07)	<.001	-4.28 (-8.06, -0.50)	0.027
**Unknown**	10.04 (0.72, 19.35)	0.035			-7.00 (-16.24, 2.24)	0.14		
**Metropolitan residency** [b]								
**Non-metropolitan**	Ref		Ref		Ref		Ref	
**Metropolitan**	1.11 (0.12, 2.10)	0.028	0.82 (-1.10, 2.75)	0.40	-0.03 (-0.87, 0.82)	0.95	0.11 (-1.68, 1.90)	0.90
**Dual eligibility** [c]								
**Non-dual**	Ref		Ref		Ref		Ref	
**Dual**	-7.38 (-8.31, -6.46)	<.001	-5.44 (-7.39, -3.49)	<.001	-2.87 (-3.75, -2.00)	<.001	-2.12 (-3.96, -0.27)	0.024
**Marital Status**								

© 2026 Hu X et al. *JAMA Network Open*.

Variables	Systemic Therapy				Hospice Enrollment			
	Without Matching		Matched		Without Matching		Matched	
	Adjusted Probability Difference (95%CI)	P-value	Adjusted Probability Difference (95%CI)	P-value	Adjusted Probability Difference (95%CI)	P-value	Adjusted Probability Difference (95%CI)	P-value
**Married**	Ref		Ref		Ref		Ref	
**Not married**	-8.05 (-8.81, -7.30)	<.001	-5.22 (-6.68, -3.76)	<.001	-0.72 (-1.40, -0.04)	0.038	0.52 (-0.90, 1.94)	0.48
**Unknown**	-5.36 (-6.31, -4.41)	<.001	-4.69 (-6.42, -2.95)	<.001	5.58 (4.78, 6.39)	<.001	6.79 (5.19, 8.38)	<.001
**Yost Quintile** [d]								
**Q1 (Lowest SES)**	Ref		Ref		Ref		Ref	
**Q2**	0.26 (-0.92, 1.43)	0.67	0.81 (-1.49, 3.12)	0.49	-0.97 (-1.96, 0.03)	0.056	-1.13 (-3.14, 0.87)	0.27
**Q3**	1.58 (0.41, 2.74)	0.008	2.59 (0.30, 4.87)	0.026	-0.66 (-1.64, 0.33)	0.19	-0.69 (-2.67, 1.29)	0.49
**Q4**	3.70 (2.54, 4.86)	<.001	4.11 (1.85, 6.37)	<.001	-0.78 (-1.77, 0.21)	0.12	-0.57 (-2.56, 1.42)	0.58
**Q5 (Highest SES)**	6.92 (5.78, 8.07)	<.001	5.68 (3.39, 7.98)	<.001	-1.27 (-2.26, -0.28)	0.012	-2.23 (-4.30, -0.17)	0.034
**Unknown**	4.38 (2.94, 5.82)	<.001	3.45 (0.72, 6.17)	0.013	-3.34 (-4.66, -2.02)	<.001	-2.83 (-5.48, -0.18)	0.036
**Cancer site**								
**Breast**	Ref		Ref		Ref		Ref	
**Colorectal**	20.95 (19.50, 22.40)	<.001	15.55 (12.73, 18.36)	<.001	-7.62 (-9.16, -6.08)	<.001	-6.90 (-10.21, -3.59)	<.001
**NSCLC**	4.25 (3.23, 5.27)	<.001	2.67 (0.71, 4.63)	0.008	-4.41 (-5.25, -3.57)	<.001	-3.82 (-5.57, -2.07)	<.001
**Pancreas**	5.23 (4.00, 6.46)	<.001	5.37 (3.03, 7.72)	<.001	6.90 (5.95, 7.86)	<.001	7.89 (5.93, 9.86)	<.001
**Prostate**	30.30 (29.16, 31.44)	<.001	21.82 (19.60, 24.04)	<.001	-6.07 (-7.33, -4.82)	<.001	-4.24 (-7.06, -1.41)	0.003
**SCLC**	17.00 (15.75, 18.25)	<.001	12.87 (10.60, 15.13)	<.001	-0.19 (-1.28, 0.90)	0.73	0.29 (-1.91, 2.49)	0.79
**NCI Comorbidity Index**	-5.62 (-6.10, -5.13)	<.001	-3.80 (-4.79, -2.82)	<.001	-1.44 (-1.88, -0.99)	<.001	-2.58 (-3.50, -1.65)	<.001

* *Notes*: MA=Medicare Advantage; NSCL = Non-Small Cell Lung; SCL = Small Cell Lung; SES = Socioeconomic Status; TM=Traditional Medicare. We conducted 1:1 exact matching on treating oncologists for MA beneficiaries under each contract to TM beneficiaries treated by the same oncologists. Standard errors were clustered to the beneficiary level in the matched cohort because some TM beneficiaries we matched to multiple MA beneficiaries.

[a] Other group includes American Indian/Alaska Native, Asian/Pacific Islander, and other unspecified.

[b] Based on 2013 Rural Urban Continuum Code. Codes 1-3 were classified as metropolitan, and Codes 4-9 were classified as non-metropolitan.

[c] With state buy-in (Medicaid) from cancer diagnosis to the end of follow-up.

[d] Yost index is a composite SES scores for census tracts based on Median household income, Median house value, Median rent, Percent below 150% of federal poverty line, Education Index, Percent working class, and Percent unemployed. Lower quintiles represent the lower SES groups.

© 2026 Hu X et al. *JAMA Network Open*.

eTable 8. Logistic Regression for Systemic Therapy Receipt and Hospice Enrollment Comparison Between Medicare Advantage Plan Types and Traditional Medicare Before and After Matching Based on Treating Oncologists

Variables	Systemic Therapy				Hospice Enrollment			
	Without Matching		Matched		Without Matching		Matched	
	Adjusted Probability Difference (95%CI)	P-value	Adjusted Probability Difference (95%CI)	P-value	Adjusted Probability Difference (95%CI)	P-value	Adjusted Probability Difference (95%CI)	P-value
**Medicare Plan Type**								
**TM**	Ref		Ref		Ref		Ref	
**HMO**	-7.32 (-8.09, -6.54)	<.001	-7.76 (-9.26, -6.26)	<.001	4.09 (3.40, 4.78)	<.001	1.49 (0.15, 2.84)	0.030
**Local PPO**	-1.27 (-2.32, -0.22)	0.018	-2.64 (-4.16, -1.12)	0.001	2.94 (1.94, 3.94)	<.001	2.23 (0.76, 3.69)	0.003
**Region PPO**	-4.51 (-6.76, -2.25)	<.001	-7.11 (-10.20, -4.02)	<.001	1.07 (-1.12, 3.27)	0.34	-0.07 (-2.90, 2.77)	0.96
**POS**	-6.78 (-9.44, -4.11)	<.001	-10.41 (-14.60, -6.23)	<.001	2.11 (-0.42, 4.64)	0.10	3.34 (-0.09, 6.77)	0.057
**Other/Unk**	-3.33 (-6.25, -0.40)	0.026	-2.30 (-6.44, 1.84)	0.28	-1.98 (-4.88, 0.93)	0.18	0.50 (-3.36, 4.37)	0.80
**Year of Diagnosis**								
**2016**	Ref		Ref		Ref		Ref	
**2017**	0.53 (-0.47, 1.52)	0.30	0.76 (-1.04, 2.55)	0.41	0.21 (-0.64, 1.06)	0.63	0.93 (-0.87, 2.73)	0.31
**2018**	1.49 (0.50, 2.48)	0.003	0.55 (-1.23, 2.33)	0.55	-0.01 (-0.87, 0.84)	0.98	1.28 (-0.51, 3.08)	0.16
**2019**	0.89 (-0.11, 1.88)	0.08	-0.20 (-1.98, 1.58)	0.83	-0.80 (-1.67, 0.08)	0.074	0.28 (-1.51, 2.07)	0.76
**2020**	-2.00 (-3.07, -0.93)	<.001	-2.31 (-4.89, 0.26)	0.079	-0.52 (-1.47, 0.42)	0.28	0.22 (-1.71, 2.14)	0.83
**2021**	-12.12 (-13.48, -10.76)	<.001	-10.27 (-13.36, -7.18)	<.001	-3.67 (-4.88, -2.47)	<.001	-4.19 (-6.88, -1.50)	0.002
**Age groups**								
**66-74**	Ref		Ref		Ref		Ref	
**75-84**	-8.12 (-8.78, -7.46)	<.001	-6.35 (-7.59, -5.11)	<.001	4.52 (3.92, 5.13)	<.001	4.30 (3.08, 5.51)	<.001
**85+**	-25.15 (-26.16, -24.13)	<.001	-19.53 (-21.80, -17.26)	<.001	9.54 (8.76, 10.33)	<.001	9.40 (7.69, 11.12)	<.001
**Sex**								
**Male**	Ref		Ref		Ref		Ref	
**Female**	-2.01 (-2.67, -1.34)	<.001	-2.56 (-3.81, -1.31)	<.001	6.00 (5.39, 6.60)	<.001	5.77 (4.56, 6.99)	<.001
**Race and ethnicity**								
**Non-Hispanic White**	Ref		Ref		Ref		Ref	
**Hispanic**	-2.04 (-3.21, -0.87)	0.001	-0.88 (-3.21, 1.44)	0.46	3.25 (2.11, 4.39)	<.001	4.78 (2.44, 7.12)	<.001
**Non-Hispanic Black**	-2.87 (-4.30, -1.45)	<.001	-0.15 (-2.90, 2.60)	0.92	-6.07 (-7.48, -4.65)	<.001	-5.36 (-8.23, -2.48)	<.001
**Non-Hispanic Other** [a]	3.09 (1.39, 4.79)	<.001	2.13 (-1.48, 5.74)	0.25	-4.81 (-6.56, -3.06)	<.001	-4.28 (-8.06, -0.50)	0.026
**Unknown**	9.66 (0.40, 18.92)	0.041			-6.89 (-16.14, 2.35)	0.14		
**Metropolitan residency** [b]								
**Non-metropolitan**	Ref		Ref		Ref		Ref	
**Metropolitan**	1.53 (0.53, 2.53)	0.003	1.17 (-0.81, 3.14)	0.25	-0.19 (-1.04, 0.66)	0.66	0.02 (-1.79, 1.84)	0.98
**Dual eligibility** [c]								

© 2026 Hu X et al. *JAMA Network Open*.

Variables	Systemic Therapy				Hospice Enrollment			
	Without Matching		Matched		Without Matching		Matched	
	Adjusted Probability Difference (95%CI)	P-value	Adjusted Probability Difference (95%CI)	P-value	Adjusted Probability Difference (95%CI)	P-value	Adjusted Probability Difference (95%CI)	P-value
**Non-dual**	Ref		Ref		Ref		Ref	
**Dual**	-7.09 (-8.01, -6.16)	<.001	-4.87 (-6.83, -2.92)	<.001	-2.91 (-3.78, -2.03)	<.001	-1.97 (-3.83, -0.11)	0.038
**Marital Status**								
**Married**	Ref		Ref		Ref		Ref	
**Not married**	-7.99 (-8.74, -7.23)	<.001	-5.03 (-6.49, -3.57)	<.001	-0.72 (-1.40, -0.04)	0.039	0.55 (-0.88, 1.97)	0.45
**Unknown**	-5.52 (-6.48, -4.57)	<.001	-4.66 (-6.42, -2.90)	<.001	5.74 (4.94, 6.54)	<.001	6.93 (5.32, 8.54)	<.001
**Yost Quintile** [d]								
**Q1 (Lowest SES)**	Ref		Ref		Ref		Ref	
**Q2**	0.24 (-0.93, 1.42)	0.69	0.72 (-1.58, 3.03)	0.54	-0.96 (-1.96, 0.03)	0.057	-1.13 (-3.14, 0.87)	0.27
**Q3**	1.63 (0.47, 2.80)	0.006	2.55 (0.27, 4.83)	0.028	-0.69 (-1.68, 0.29)	0.17	-0.71 (-2.69, 1.27)	0.48
**Q4**	3.79 (2.63, 4.95)	<.001	4.16 (1.90, 6.41)	<.001	-0.84 (-1.83, 0.15)	0.098	-0.59 (-2.58, 1.41)	0.57
**Q5 (Highest SES)**	7.06 (5.92, 8.21)	<.001	5.72 (3.43, 8.00)	<.001	-1.35 (-2.34, -0.35)	0.008	-2.25 (-4.32, -0.19)	0.032
**Unknown**	4.20 (2.75, 5.65)	<.001	3.00 (0.23, 5.77)	0.034	-3.47 (-4.80, -2.15)	<.001	-3.10 (-5.79, -0.42)	0.024
**Cancer site**								
**Breast**	Ref		Ref		Ref		Ref	
**Colorectal**	20.97 (19.53, 22.42)	<.001	15.56 (12.74, 18.37)	<.001	-7.62 (-9.16, -6.08)	<.001	-6.93 (-10.24, -3.62)	<.001
**NSCLC**	4.27 (3.25, 5.28)	<.001	2.70 (0.74, 4.66)	0.007	-4.41 (-5.24, -3.57)	<.001	-3.83 (-5.58, -2.08)	<.001
**Pancreas**	5.20 (3.97, 6.43)	<.001	5.41 (3.07, 7.76)	<.001	6.92 (5.96, 7.87)	<.001	7.88 (5.91, 9.84)	<.001
**Prostate**	30.35 (29.22, 31.49)	<.001	21.83 (19.61, 24.05)	<.001	-6.11 (-7.36, -4.85)	<.001	-4.26 (-7.09, -1.43)	0.003
**SCLC**	16.98 (15.73, 18.23)	<.001	12.89 (10.62, 15.15)	<.001	-0.16 (-1.25, 0.92)	0.77	0.28 (-1.92, 2.48)	0.80
**NCI Comorbidity Index**	-5.57 (-6.05, -5.09)	<.001	-3.79 (-4.77, -2.81)	<.001	-1.44 (-1.89, -1.00)	<.001	-2.56 (-3.49, -1.64)	<.001

* *Notes*: HMO = Health Maintenance Organization; NSCL = Non-Small Cell Lung; SCL = Small Cell Lung; PPO = Preferred Provider Organization; POS = Point of Service; SES = Socioeconomic Status; TM=Traditional Medicare. We conducted 1:1 exact matching on treating oncologists for MA beneficiaries under each contract to TM beneficiaries treated by the same oncologists. Standard errors were clustered to the beneficiary level in the matched cohort because some TM beneficiaries we matched to multiple MA beneficiaries.

[a] Other group includes American Indian/Alaska Native, Asian/Pacific Islander, and other unspecified.

[b] Based on 2013 Rural Urban Continuum Code. Codes 1-3 were classified as metropolitan, and Codes 4-9 were classified as non-metropolitan.

[c] With state buy-in (Medicaid) from cancer diagnosis to the end of follow-up.

[d] Yost index is a composite SES scores for census tracts based on Median household income, Median house value, Median rent, Percent below 150% of federal poverty line, Education Index, Percent working class, and Percent unemployed. Lower quintiles represent the lower SES groups.

© 2026 Hu X et al. *JAMA Network Open*.

eFigure 4. Cumulative Incidence of Palliative Care up to 6 Months Before and After Matching Based on Treating Oncologists Among Beneficiaries with ≥6 months' survival

A. Overall Sample by Medicare Advantage and Traditional Medicare

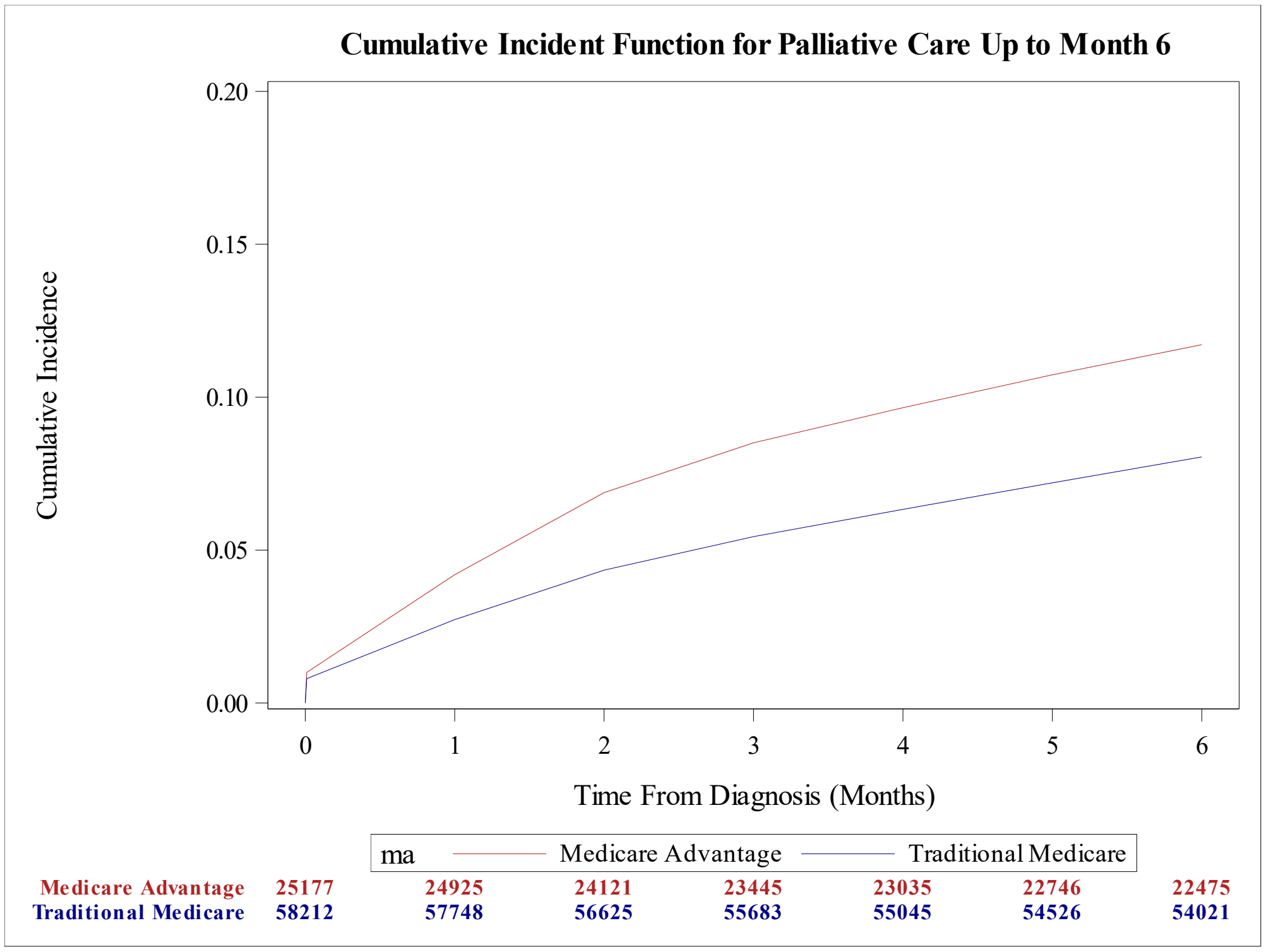

B. Among Matched and Unmatched Medicare Advantage and Traditional Medicare Cohorts

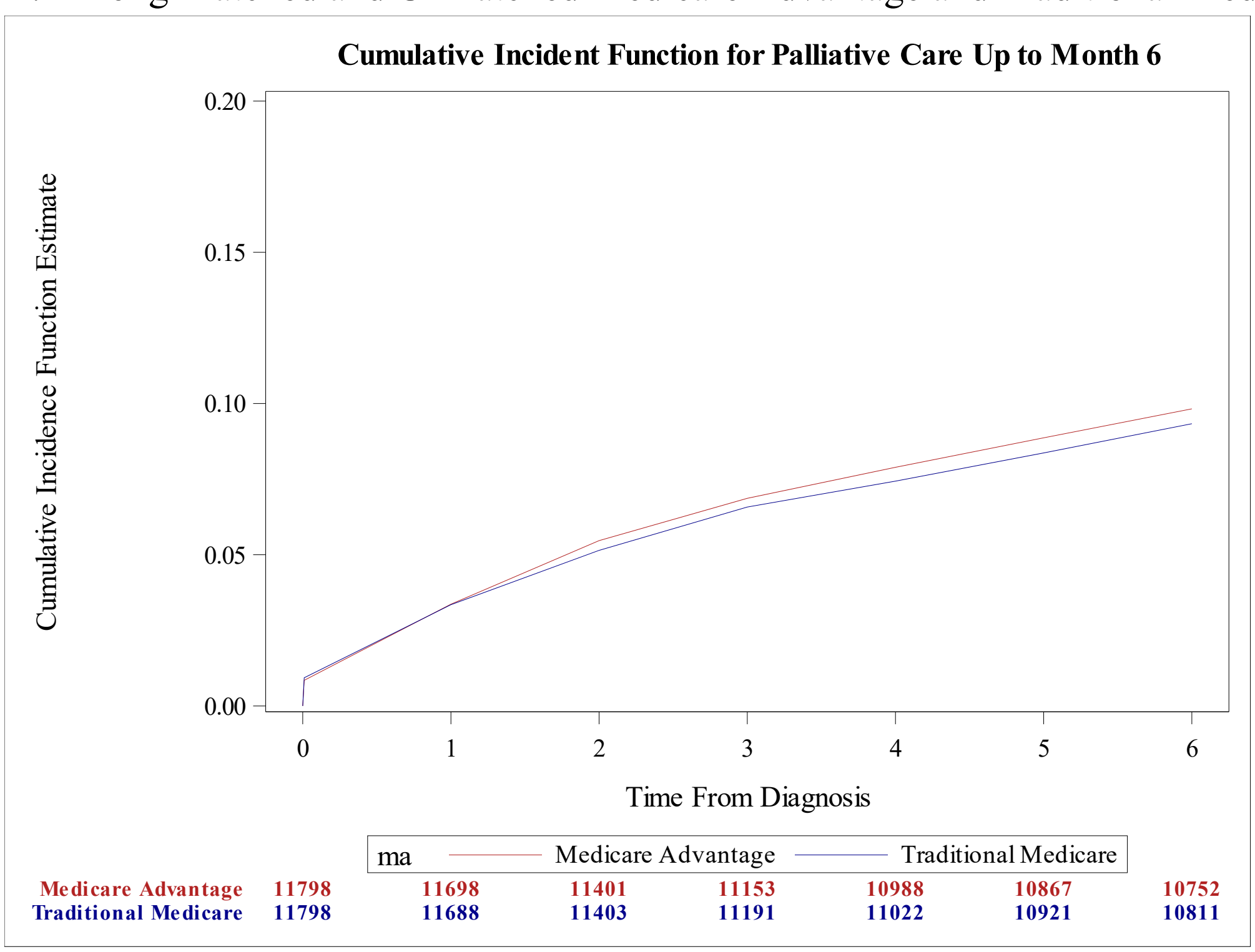

© 2026 Hu X et al. *JAMA Network Open*.

eFigure 5. Cumulative Incidence of Palliative Care up to 6 Months Before and After Matching Based on Treating Oncologists Among Beneficiaries Diagnosed and Died Outside COVID-19 Disruption Period

A. Overall Sample by Medicare Advantage and Traditional Medicare

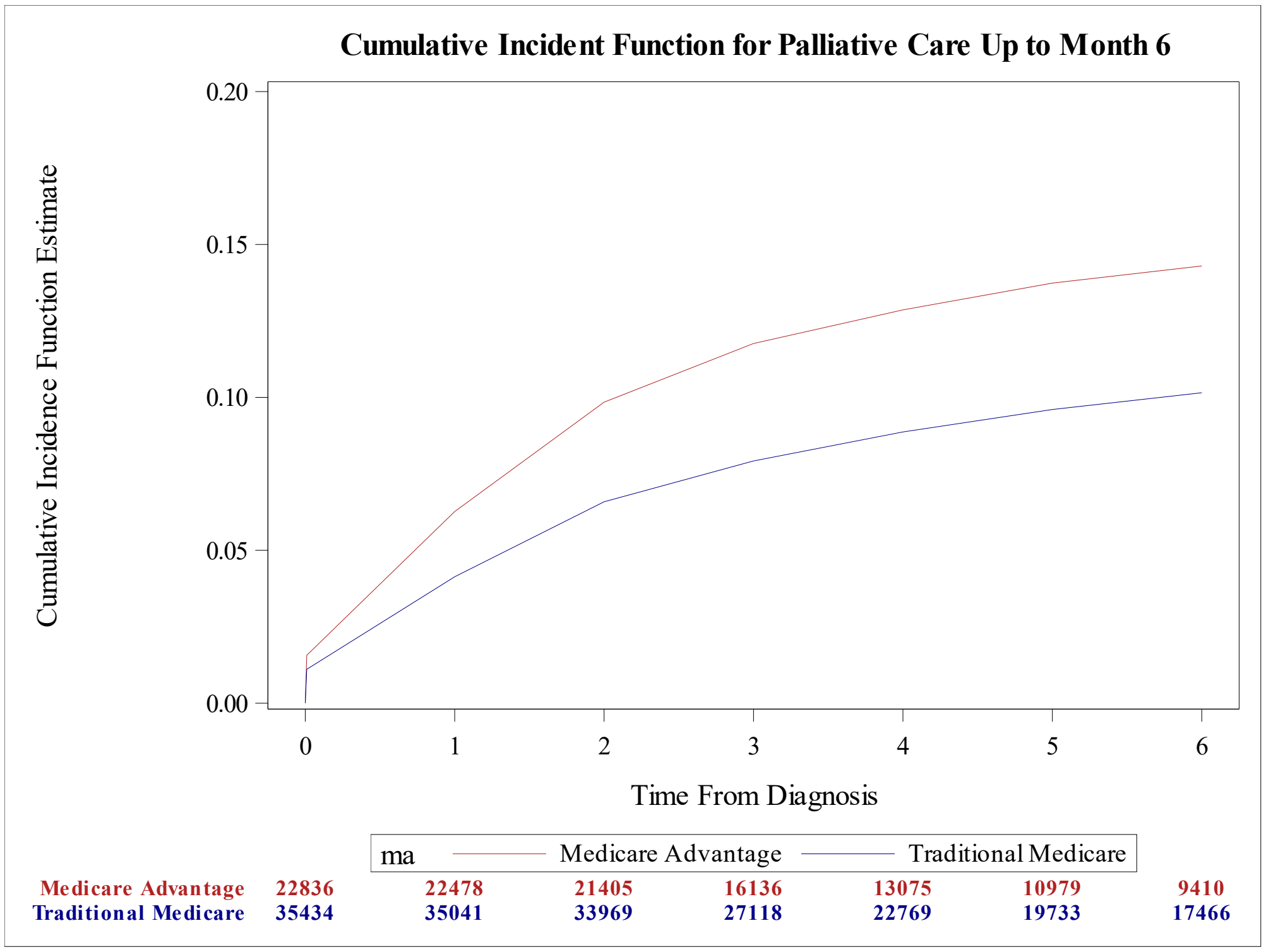

B. Among Matched and Unmatched Medicare Advantage and Traditional Medicare Cohorts

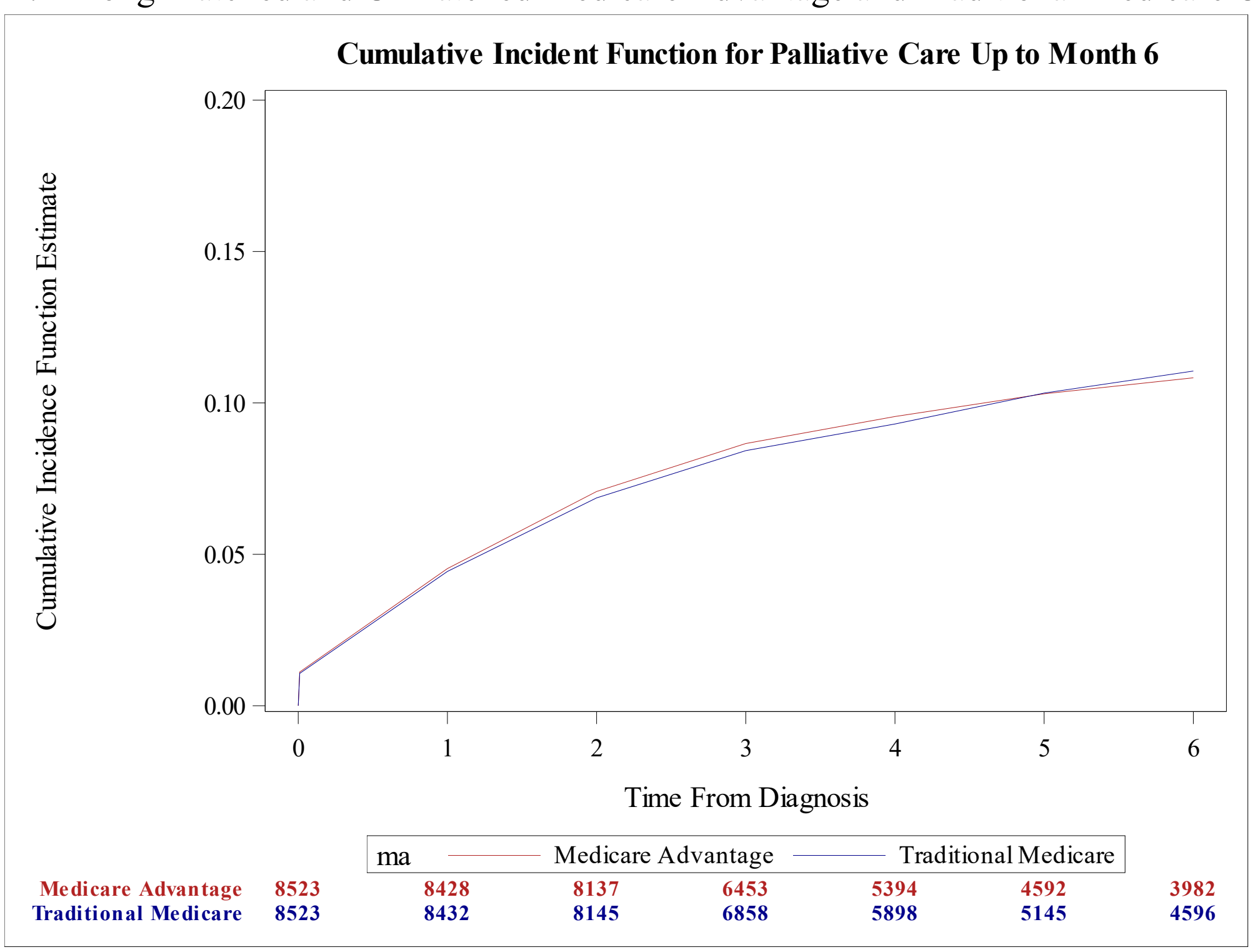

© 2026 Hu X et al. *JAMA Network Open*.

eTable 9. Cox Proportional Hazards Regression Model for Palliative Care Receipt up to 6 Months Before and After Matching Based on Treating Oncologists Among Beneficiaries with ≥6 months' survival

Variables	Without Matching		Matched	
	HR (95%CI)	P-value	HR (95%CI)	P-value
**Total N**	**83,389**		**23,596**	
**Medicare Plan Type**				
**TM**	Ref		Ref	
**MA**	1.40 (1.34, 1.47)	<.001	1.04 (0.96, 1.13)	0.31
**Year of Diagnosis**				
**2016**	Ref		Ref	
**2017**	1.22 (1.12, 1.34)	<.001	1.14 (0.95, 1.37)	0.17
**2018**	1.60 (1.47, 1.74)	<.001	1.43 (1.21, 1.69)	<.001
**2019**	1.70 (1.57, 1.85)	<.001	1.64 (1.40, 1.92)	<.001
**2020**	1.68 (1.55, 1.83)	<.001	1.58 (1.35, 1.85)	<.001
**2021**	1.93 (1.76, 2.11)	<.001	1.97 (1.65, 2.34)	<.001
**Age groups**				
**66-74**	Ref		Ref	
**75-84**	0.92 (0.88, 0.97)	0.001	0.91 (0.84, 1.00)	0.042
**85+**	0.91 (0.84, 0.98)	0.012	0.99 (0.86, 1.15)	0.91
**Sex**				
**Male**	Ref		Ref	
**Female**	1.07 (1.01, 1.12)	0.014	1.01 (0.93, 1.11)	0.75
**Race and ethnicity**				
**Non-Hispanic White**	Ref		Ref	
**Hispanic**	1.20 (1.11, 1.31)	<.001	1.16 (0.99, 1.36)	0.076
**Non-Hispanic Black**	1.06 (0.98, 1.15)	0.17	0.98 (0.84, 1.13)	0.76
**Non-Hispanic Other** [a]	1.09 (0.99, 1.20)	0.084	1.03 (0.85, 1.26)	0.76
**Unknown**	0.91 (0.53, 1.57)	0.74	0.78 (0.24, 2.54)	0.68
**Metropolitan residency** [b]				
**Non-metropolitan**	Ref		Ref	
**Metropolitan**	1.17 (1.08, 1.27)	<.001	1.18 (1.02, 1.37)	0.029
**Dual eligibility** [c]				
**Non-dual**	Ref		Ref	
**Dual**	1.06 (0.99, 1.15)	0.108	1.25 (1.09, 1.42)	0.001
**Marital Status**				
**Married**	Ref		Ref	
**Not married**	1.08 (1.02, 1.14)	0.006	1.12 (1.02, 1.23)	0.023
**Unknown**	0.81 (0.75, 0.87)	<.001	0.79 (0.69, 0.91)	0.001
**Yost Quintile** [d]				
**Q1 (Lowest SES)**	Ref		Ref	
**Q2**	1.19 (1.08, 1.31)	<.001	0.97 (0.83, 1.14)	0.72
**Q3**	1.23 (1.12, 1.35)	<.001	1.06 (0.91, 1.23)	0.49
**Q4**	1.38 (1.26, 1.51)	<.001	1.09 (0.94, 1.27)	0.27
**Q5 (Highest SES)**	1.63 (1.49, 1.78)	<.001	1.27 (1.09, 1.48)	0.002
**Unknown**	1.14 (1.01, 1.29)	0.041	0.86 (0.69, 1.08)	0.20
**Cancer site**				
**Breast**	Ref		Ref	
**Colorectal**	0.87 (0.78, 0.97)	0.015	0.83 (0.66, 1.05)	0.12
**NSCLC**	1.43 (1.33, 1.53)	<.001	1.64 (1.43, 1.88)	<.001
**Pancreas**	2.14 (1.97, 2.33)	<.001	2.29 (1.95, 2.70)	<.001
**Prostate**	0.60 (0.54, 0.67)	<.001	0.62 (0.50, 0.77)	<.001
**SCLC**	1.48 (1.35, 1.63)	<.001	1.60 (1.34, 1.90)	<.001
**NCI Comorbidity Index**	0.99 (0.95, 1.03)	0.73	0.98 (0.91, 1.05)	0.54

* *Notes*: MA = Medicare Advantage; NSCL = Non-Small Cell Lung; SCL = Small Cell Lung; SES = Socioeconomic Status; TM = Traditional Medicare. We conducted 1:1 exact matching on treating oncologists for MA beneficiaries

© 2026 Hu X et al. *JAMA Network Open*.

under each contract to TM beneficiaries treated by the same oncologists. Standard errors were clustered to the beneficiary level in the matched cohort because some TM beneficiaries we matched to multiple MA beneficiaries.

[a] Other group includes American Indian/Alaska Native, Asian/Pacific Islander, and other unspecified.

[b] Based on 2013 Rural Urban Continuum Code. Codes 1-3 were classified as metropolitan, and Codes 4-9 were classified as non-metropolitan.

[c] With state buy-in (Medicaid) from cancer diagnosis to the end of follow-up.

[d] Yost index is a composite SES scores for census tracts based on Median household income, Median house value, Median rent, Percent below 150% of federal poverty line, Education Index, Percent working class, and Percent unemployed. Lower quintiles represent the lower SES groups.

© 2026 Hu X et al. *JAMA Network Open*.

eTable 10. Cox Proportional Hazards Regressino Model for Palliative Care Receipt up to 6 Months Before and After Matching Based on Treating Oncologists Among Beneficiaries Diagnosed and Died Outside COVID-19 Disruption Period

Variables	Without Matching		Matched	
	**HR (95%CI)**	**P-value**	**HR (95%CI)**	**P-value**
Total N	**51,408**		**17,062**	
**Medicare Plan Type**				
**TM**	Ref		Ref	
**MA**	1.36 (1.30, 1.43)	<.001	0.97 (0.89, 1.06)	0.54
**Year of Diagnosis**				
**2016**	Ref		Ref	
**2017**	1.23 (1.14, 1.33)	<.001	1.15 (1.00, 1.32)	0.056
**2018**	1.55 (1.44, 1.67)	<.001	1.46 (1.27, 1.67)	<.001
**2019**	1.69 (1.56, 1.82)	<.001	1.69 (1.46, 1.94)	<.001
**2020**	1.06 (0.81, 1.37)	0.68	----	
**2021**	2.11 (1.94, 2.29)	<.001	2.06 (1.73, 2.44)	<.001
**Age groups**				
**66-74**	Ref		Ref	
**75-84**	0.90 (0.86, 0.95)	<.001	0.86 (0.78, 0.95)	0.002
**85+**	0.88 (0.82, 0.95)	0.001	0.86 (0.73, 1.00)	0.054
**Sex**				
**Male**	Ref		Ref	
**Female**	1.03 (0.98, 1.08)	0.27	0.95 (0.86, 1.05)	0.31
**Race and ethnicity**				
**Non-Hispanic White**	Ref		Ref	
**Hispanic**	1.16 (1.07, 1.27)	0.001	1.20 (1.00, 1.43)	0.049
**Non-Hispanic Black**	0.94 (0.86, 1.03)	0.18	1.00 (0.85, 1.17)	0.99
**Non-Hispanic Other** [a]	1.10 (0.98, 1.22)	0.099	0.91 (0.70, 1.17)	0.46
**Unknown**	1.07 (0.52, 2.21)	0.86	----	
**Metropolitan residency** [b]				
**Non-metropolitan**	Ref		Ref	
**Metropolitan**	1.11 (1.02, 1.20)	0.015	1.15 (0.99, 1.34)	0.070
**Dual eligibility** [c]				
**Non-dual**	Ref		Ref	
**Dual**	0.98 (0.91, 1.05)	0.51	1.04 (0.90, 1.19)	0.63
**Marital Status**				
**Married**	Ref		Ref	
**Not married**	1.07 (1.01, 1.13)	0.021	1.12 (1.01, 1.25)	0.036
**Unknown**	0.81 (0.75, 0.88)	<.001	0.89 (0.77, 1.03)	0.11
**Yost Quintile** [d]				
**Q1 (Lowest SES)**	Ref		Ref	
**Q2**	1.09 (0.99, 1.20)	0.068	0.88 (0.73, 1.05)	0.14
**Q3**	1.19 (1.08, 1.30)	<.001	1.22 (1.03, 1.43)	0.021
**Q4**	1.26 (1.15, 1.39)	<.001	1.25 (1.06, 1.48)	0.007
**Q5 (Highest SES)**	1.52 (1.39, 1.66)	<.001	1.28 (1.08, 1.51)	0.004
**Unknown**	1.28 (1.13, 1.44)	<.001	1.08 (0.87, 1.36)	0.48
**Cancer site**				
**Breast**	Ref		Ref	
**Colorectal**	0.97 (0.84, 1.11)	0.66	0.86 (0.64, 1.16)	0.32
**NSCLC**	1.28 (1.18, 1.38)	<.001	1.43 (1.22, 1.67)	<.001
**Pancreas**	1.60 (1.47, 1.75)	<.001	1.78 (1.49, 2.12)	<.001
**Prostate**	0.77 (0.67, 0.88)	0.001	0.66 (0.48, 0.91)	0.011
**SCLC**	1.17 (1.06, 1.30)	0.003	1.30 (1.07, 1.58)	0.009
**NCI Comorbidity Index**	0.93 (0.89, 0.96)	0.001	0.96 (0.89, 1.04)	0.33

© 2026 Hu X et al. *JAMA Network Open*.

* *Notes*: MA = Medicare Advantage; NSCL = Non-Small Cell Lung; SCL = Small Cell Lung; SES = Socioeconomic Status; TM = Traditional Medicare. Standard errors were clustered to the beneficiary level in the matched cohort because some TM beneficiaries we matched to multiple MA beneficiaries.
[a] Other group includes American Indian/Alaska Native, Asian/Pacific Islander, and other unspecified.
[b] Based on 2013 Rural Urban Continuum Code. Codes 1-3 were classified as metropolitan, and Codes 4-9 were classified as non-metropolitan.
[c] With state buy-in (Medicaid) from cancer diagnosis to the end of follow-up.
[d] Yost index is a composite SES scores for census tracts based on Median household income, Median house value, Median rent, Percent below 150% of federal poverty line, Education Index, Percent working class, and Percent unemployed. Lower quintiles represent the lower SES groups.

© 2026 Hu X et al. *JAMA Network Open*.

eFigure 6. Cox Proportional Hazards Regression Model Comparing Palliative Care Receipt up to 6 Months Between Medicare Advantage and Traditional Medicare Before and After Matching Based on Treating Oncologists by Cancer Site

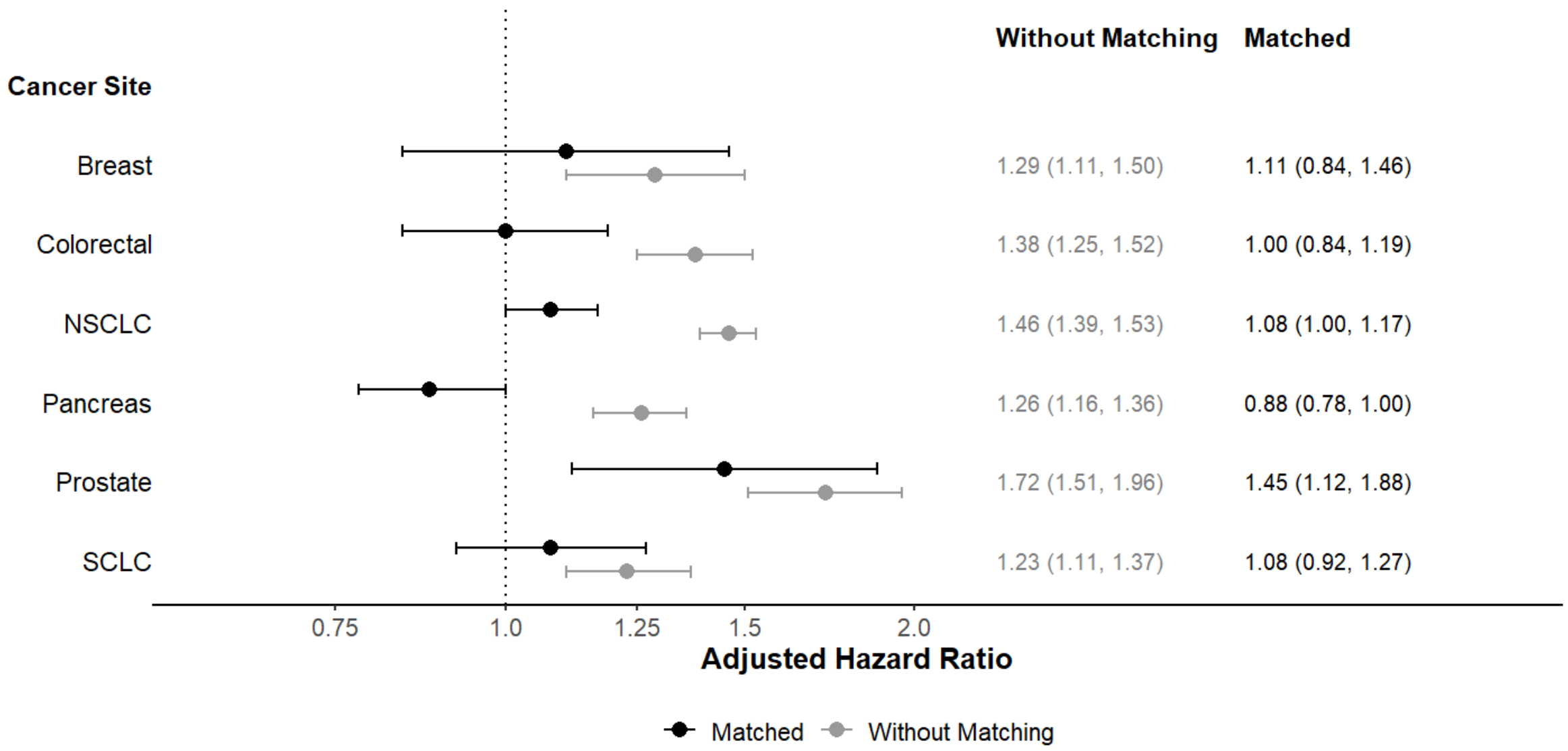

* *Notes*: NSCL = Non-Small Cell Lung; SCL = Small Cell Lung. We conducted 1:1 exact matching on treating oncologists for MA beneficiaries under each contract to TM beneficiaries treated by the same oncologists. Adjusted probability differences estimated from regression models with an interaction term between cancer sites and Medicare Advantage vs. Traditional Medicare indicator, adjusting for patients' age, sex, race and ethnicity, metropolitan residency, marital status, dual eligibility, marital status, Yost index quantile, cancer site, year of diagnosis, and NCI comorbidity index. Standard errors were clustered to the beneficiary level in the matched cohort because some TM beneficiaries we matched to multiple MA beneficiaries.

© 2026 Hu X et al. *JAMA Network Open*.

eFigure 7. Systemic Therapy Receipt and Hospice Enrollment Comparison Between Medicare Advantage and Traditional Medicare Before and After Matching Based on Treating Oncologists by Cancer Site

A. Systemic Therapy by Cancer Site

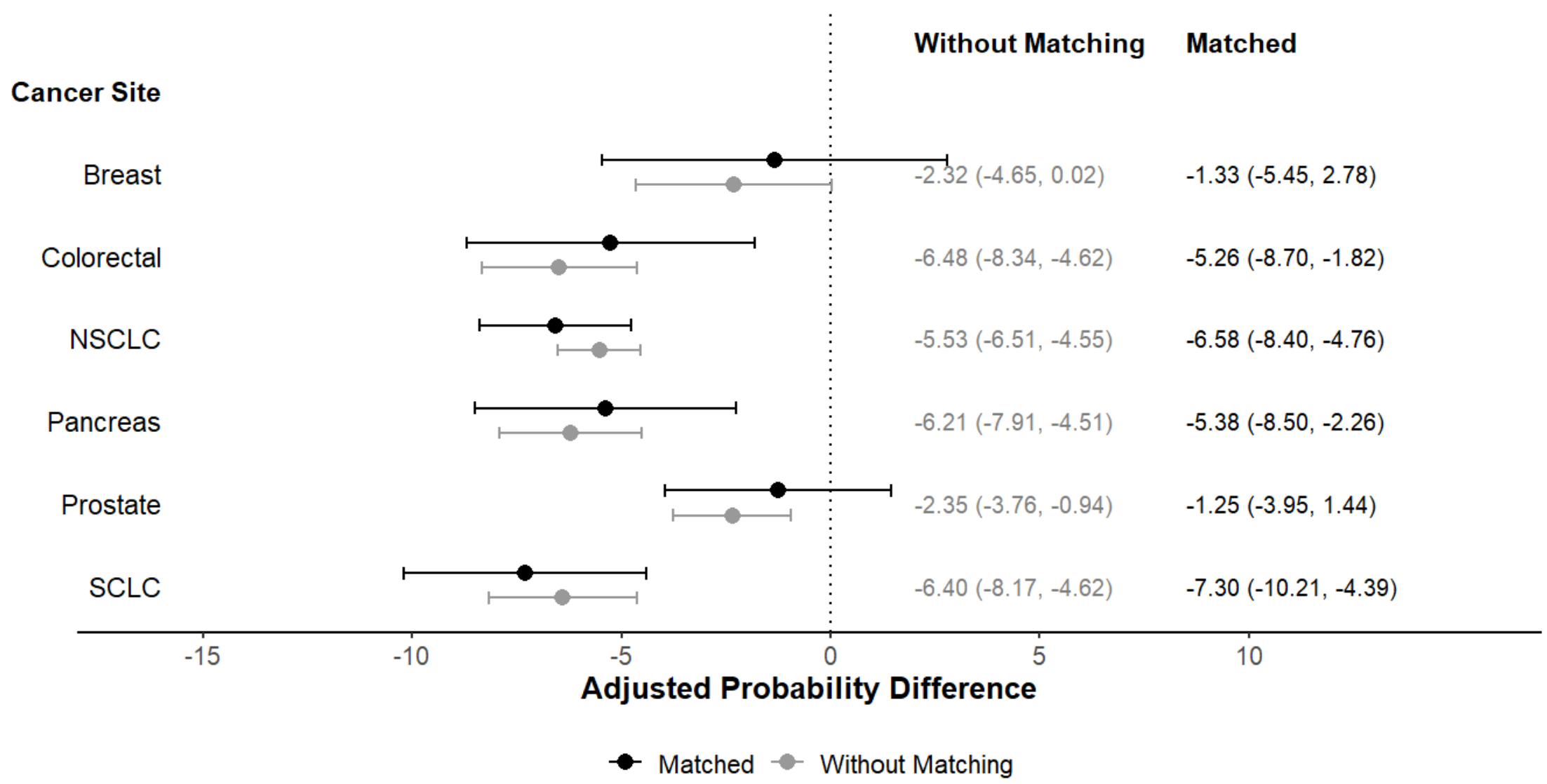

B. Hospice Enrollment by Cancer Site

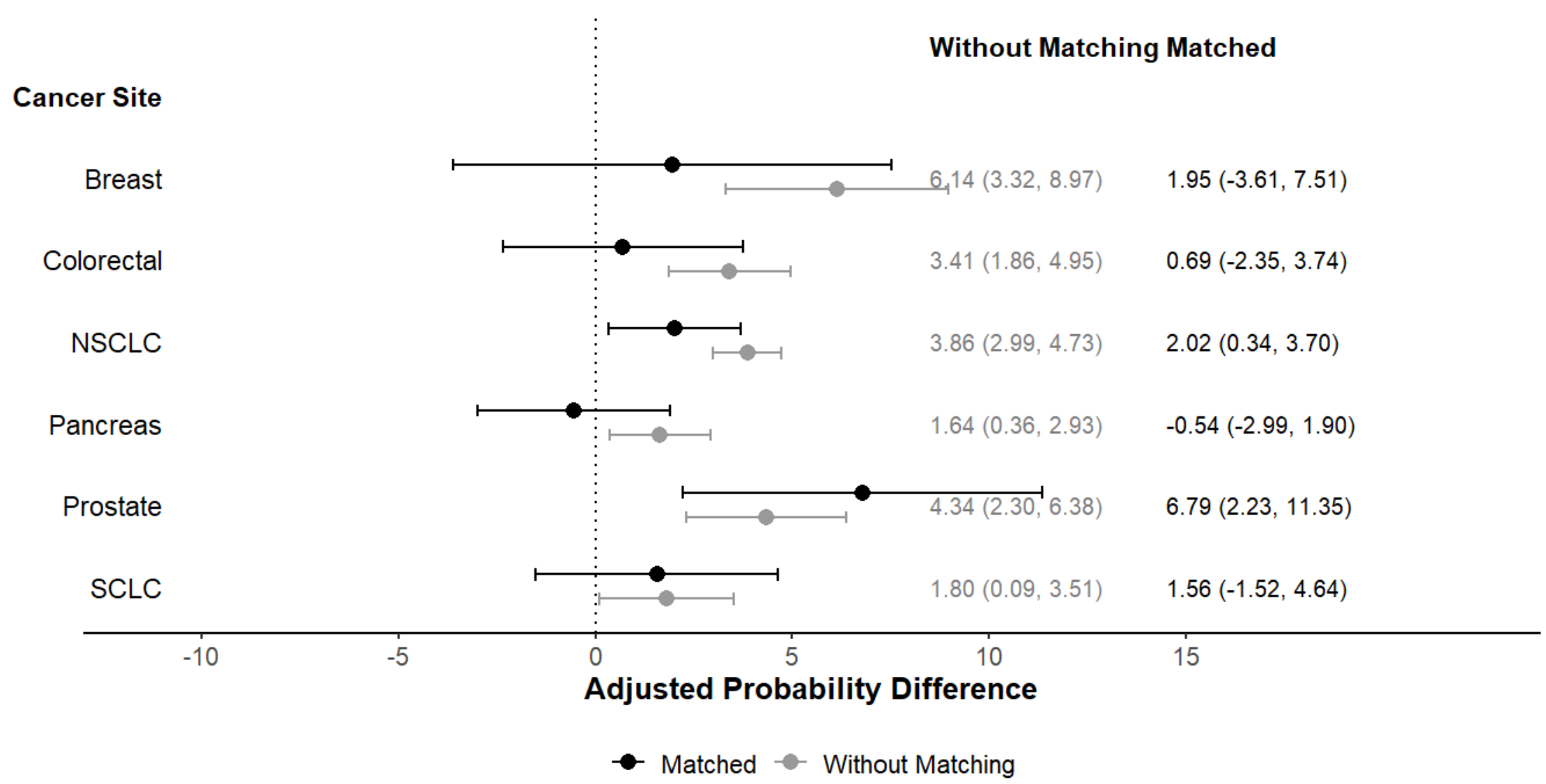

* *Notes*: NSCL = Non-Small Cell Lung; SCL = Small Cell Lung. We conducted 1:1 exact matching on treating oncologists for MA beneficiaries under each contract to TM beneficiaries treated by the same oncologists. Adjusted probability differences estimated from regression models with an interaction term between cancer sites and Medicare Advantage vs. Traditional Medicare indicator, adjusting for patients' age, sex, race and ethnicity, metropolitan residency, marital status, dual eligibility, marital status, Yost index quantile, cancer site, year of diagnosis, and NCI comorbidity index. Standard errors were clustered to the beneficiary level in the matched cohort because some TM beneficiaries we matched to multiple MA beneficiaries.

© 2026 Hu X et al. *JAMA Network Open*.

eTable 11. Cox Proportional Hazards Regression Model for Palliative Care Comparison Between Medicare Advantage and Traditional Medicare Among Plans with High Data Completeness and without 100% Missing Provider IDs in Outpatient and Carrier Files

Variables	HR (95%CI)	P-value
**Total N**	**117,069**	
**Medicare Plan Type**		
**TM**	Ref	
**MA**	1.12 (1.07, 1.17)	<.001
**Year of Diagnosis**		
**2016**	Ref	
**2017**	1.23 (1.14, 1.33)	<.001
**2018**	1.48 (1.38, 1.59)	<.001
**2019**	1.66 (1.54, 1.78)	<.001
**2020**	1.63 (1.52, 1.75)	<.001
**2021**	2.08 (1.94, 2.23)	<.001
**Age groups**		
**66-74**	Ref	
**75-84**	0.92 (0.88, 0.95)	<.001
**85+**	0.91 (0.86, 0.97)	0.003
**Sex**		
**Male**	Ref	
**Female**	1.08 (1.03, 1.12)	<.001
**Race and ethnicity**		
**Non-Hispanic White**	Ref	
**Hispanic**	1.15 (1.07, 1.24)	<.001
**Non-Hispanic Black**	1.00 (0.93, 1.07)	0.95
**Non-Hispanic Other** [a]	1.03 (0.94, 1.13)	0.50
**Unknown**	0.86 (0.54, 1.36)	0.51
**Metropolitan residency** [b]		
**Non-metropolitan**	Ref	
**Metropolitan**	1.08 (1.02, 1.15)	0.015
**Dual eligibility** [c]		
**Non-dual**	Ref	
**Dual**	1.09 (1.02, 1.15)	0.006
**Marital Status**		
**Married**	Ref	
**Not married**	1.09 (1.04, 1.14)	<.001
**Unknown**	0.83 (0.79, 0.88)	<.001
**Yost Quintile** [d]		
**Q1 (Lowest SES)**	Ref	
**Q2**	1.10 (1.02, 1.19)	0.014
**Q3**	1.22 (1.13, 1.31)	<.001
**Q4**	1.28 (1.19, 1.38)	<.001
**Q5 (Highest SES)**	1.53 (1.42, 1.64)	<.001
**Unknown**	1.31 (1.20, 1.44)	<.001
**Cancer site**		
**Breast**	Ref	
**Colorectal**	0.86 (0.78, 0.95)	0.003
**NSCLC**	1.36 (1.28, 1.45)	<.001
**Pancreas**	1.91 (1.78, 2.04)	<.001

© 2026 Hu X et al. *JAMA Network Open*.

Variables	HR (95%CI)	P-value
**Prostate**	0.56 (0.51, 0.62)	<.001
**SCLC**	1.42 (1.31, 1.54)	<.001
**NCI Comorbidity Index**	0.96 (0.93, 0.99)	0.006

* *Notes*: MA = Medicare Advantage; NSCL = Non-Small Cell Lung; SCL = Small Cell Lung; SES = Socioeconomic Status; TM = Traditional Medicare. Standard errors were clustered to the beneficiary level in the regression.

[a] Other group includes American Indian/Alaska Native, Asian/Pacific Islander, and other unspecified.

[b] Based on 2013 Rural Urban Continuum Code. Codes 1-3 were classified as metropolitan, and Codes 4-9 were classified as non-metropolitan.

[c] With state buy-in (Medicaid) from cancer diagnosis to the end of follow-up.

[d] Yost index is a composite SES scores for census tracts based on Median household income, Median house value, Median rent, Percent below 150% of federal poverty line, Education Index, Percent working class, and Percent unemployed. Lower quintiles represent the lower SES groups.

© 2026 Hu X et al. *JAMA Network Open*.

eTable 12. Cox Proportional Hazards Regerssion Model for Palliative Care Comparison Between Medicare Advantage and Traditional Medicare With and Without Controlling for NCI Comorbidity Index

Variables	Without comorbidities		With comorbidities	
	HR (95%CI)	P-value	HR (95%CI)	P-value
Total N	135402		135402	
**Medicare Plan Type**				
**TM**	Ref		Ref	
**MA**	1.39 (1.34, 1.44)	<.001	1.39 (1.35, 1.44)	<.001
**Year of Diagnosis**				
**2016**	Ref		Ref	
**2017**	1.22 (1.14, 1.30)	<.001	1.22 (1.14, 1.30)	<.001
**2018**	1.47 (1.39, 1.57)	<.001	1.48 (1.39, 1.57)	<.001
**2019**	1.61 (1.51, 1.71)	<.001	1.61 (1.52, 1.71)	<.001
**2020**	1.60 (1.50, 1.70)	<.001	1.60 (1.50, 1.70)	<.001
**2021**	1.80 (1.69, 1.91)	<.001	1.80 (1.69, 1.91)	<.001
**Age groups**				
**66-74**	Ref		Ref	
**75-84**	0.94 (0.91, 0.97)	<.001	0.94 (0.91, 0.97)	0.001
**85+**	0.95 (0.90, 1.00)	0.037	0.95 (0.90, 1.00)	0.061
**Sex**				
**Male**	Ref		Ref	
**Female**	1.07 (1.03, 1.11)	<.001	1.07 (1.03, 1.11)	<.001
**Race and ethnicity**				
**Non-Hispanic White**	Ref		Ref	
**Hispanic**	1.15 (1.08, 1.23)	<.001	1.15 (1.08, 1.23)	<.001
**Non-Hispanic Black**	0.99 (0.93, 1.05)	0.75	0.99 (0.93, 1.06)	0.81
**Non-Hispanic Other** [a]	1.11 (1.03, 1.19)	0.007	1.10 (1.03, 1.19)	0.008
**Unknown**	0.84 (0.55, 1.29)	0.44	0.84 (0.55, 1.29)	0.43
**Metropolitan residency** [b]				
**Non-metropolitan**	Ref		Ref	
**Metropolitan**	1.10 (1.04, 1.17)	0.001	1.10 (1.04, 1.17)	0.001
**Dual eligibility** [c]				
**Non-dual**	Ref		Ref	
**Dual**	1.04 (0.98, 1.09)	0.19	1.04 (0.99, 1.10)	0.14
**Marital Status**				
**Married**	Ref		Ref	
**Not married**	1.06 (1.02, 1.11)	0.002	1.06 (1.02, 1.11)	0.002
**Unknown**	0.78 (0.73, 0.82)	<.001	0.78 (0.74, 0.82)	<.001
**Yost Quintile** [d]				
**Q1 (Lowest SES)**	Ref		Ref	
**Q2**	1.14 (1.06, 1.22)	<.001	1.13 (1.06, 1.21)	<.001
**Q3**	1.23 (1.15, 1.31)	<.001	1.23 (1.15, 1.31)	<.001
**Q4**	1.33 (1.24, 1.42)	<.001	1.33 (1.24, 1.42)	<.001
**Q5 (Highest SES)**	1.60 (1.50, 1.71)	<.001	1.60 (1.50, 1.70)	<.001
**Unknown**	1.33 (1.22, 1.45)	<.001	1.33 (1.22, 1.45)	<.001
**Cancer site**				
**Breast**	Ref		Ref	
**Colorectal**	0.84 (0.77, 0.92)	<.001	0.84 (0.76, 0.91)	<.001
**NSCLC**	1.35 (1.28, 1.42)	<.001	1.35 (1.28, 1.43)	<.001
**Pancreas**	1.85 (1.74, 1.97)	<.001	1.86 (1.75, 1.97)	<.001
**Prostate**	0.58 (0.54, 0.63)	<.001	0.58 (0.54, 0.63)	<.001
**SCLC**	1.35 (1.25, 1.44)	<.001	1.35 (1.26, 1.45)	<.001
**NCI Comorbidity Index**			0.98 (0.95, 1.00)	0.081

* *Notes*: MA = Medicare Advantage; NSCL = Non-Small Cell Lung; SCL = Small Cell Lung; SES = Socioeconomic Status; TM = Traditional Medicare. Standard errors were clustered to the beneficiary level in the regression.

© 2026 Hu X et al. *JAMA Network Open*.

[a] Other group includes American Indian/Alaska Native, Asian/Pacific Islander, and other unspecified.
[b] Based on 2013 Rural Urban Continuum Code. Codes 1-3 were classified as metropolitan, and Codes 4-9 were classified as non-metropolitan.
[c] With state buy-in (Medicaid) from cancer diagnosis to the end of follow-up.
[d] Yost index is a composite SES scores for census tracts based on Median household income, Median house value, Median rent, Percent below 150% of federal poverty line, Education Index, Percent working class, and Percent unemployed. Lower quintiles represent the lower SES groups.

© 2026 Hu X et al. *JAMA Network Open*.

eTable 13. Sample Characteristics With and Without Propensity Score Weighting for Beneficiary Level Characteristics

Variable	Before Weighting			Post Weighting		
	TM	MA	SMD	TM	MA	SMD
**Total N**	91,628	43,774		135,349	135,664	
**Year of Diagnosis, N (Col %)**						
**2016**	16,183 (17.7%)	5,748 (13.1%)	-0.126	21,963 (16.2%)	22,178 (16.3%)	0.003
**2017**	16,097 (17.6%)	6,146 (14.0%)	-0.097	22,235 (16.4%)	22,185 (16.4%)	-0.002
**2018**	16,142 (17.6%)	6,864 (15.7%)	-0.052	22,964 (17.0%)	22,743 (16.8%)	-0.005
**2019**	16,195 (17.7%)	8,182 (18.7%)	0.026	24,373 (18.0%)	24,504 (18.1%)	0.001
**2020**	14,466 (15.8%)	8,225 (18.8%)	0.079	22,666 (16.7%)	22,748 (16.8%)	0.001
**2021**	12,545 (13.7%)	8,609 (19.7%)	0.161	21,149 (15.6%)	21,307 (15.7%)	0.002
**Age groups, N (Col %)**						
**66-74**	41,749 (45.6%)	19,641 (44.9%)	-0.014	61,416 (45.4%)	61,605 (45.4%)	0.001
**75-84**	36,838 (40.2%)	18,190 (41.6%)	0.027	54,983 (40.6%)	55,136 (40.6%)	0.000
**85+**	13,041 (14.2%)	5,943 (13.6%)	-0.019	18,951 (14.0%)	18,923 (13.9%)	-0.002
**Sex, N (Col %)**						
**Male**	48,786 (53.2%)	23,396 (53.4%)	0.004	72,153 (53.3%)	72,332 (53.3%)	0.000
**Female**	42,842 (46.8%)	20,378 (46.6%)	-0.004	63,196 (46.7%)	63,332 (46.7%)	0.000
**Race and ethnicity, N (Col %)**						
**Hispanic**	5,116 (5.6%)	4,820 (11.0%)	0.198	9,870 (7.3%)	9,841 (7.3%)	-0.001
**Non-Hispanic Black**	7,027 (7.7%)	5,787 (13.2%)	0.182	12,782 (9.4%)	12,766 (9.4%)	-0.001
**Non-Hispanic Other** [a]	3,853 (4.2%)	2,599 (5.9%)	0.079	6,449 (4.8%)	6,422 (4.7%)	-0.001
**Non-Hispanic White**	75,459 (82.4%)	30,491 (69.7%)	-0.301	106,000 (78.3%)	106,393 (78.4%)	0.003
**Unknown**	173 (0.2%)	77 (0.2%)	-0.003	249 (0.2%)	242 (0.2%)	-0.001
**Metropolitan residency** [b], **N (Col %)**						
**Non-metropolitan**	14,956 (16.3%)	3,535 (8.1%)	-0.254	18,504 (13.7%)	18,861 (13.9%)	0.007
**Metropolitan**	76,672 (83.7%)	40,239 (91.9%)	0.254	116,845 (86.3%)	116,804 (86.1%)	-0.007
**Dual eligibility** [c], **N (Col %)**						
**Non-dual**	80,503 (87.9%)	37,940 (86.7%)	-0.036	118,377 (87.5%)	118,577 (87.4%)	-0.002
**Dual**	11,125 (12.1%)	5,834 (13.3%)	0.036	16,973 (12.5%)	17,087 (12.6%)	0.002
**Marital Status, N (Col %)**						
**Married**	36,432 (39.8%)	18,786 (42.9%)	0.064	55,168 (40.8%)	55,106 (40.6%)	-0.003
**Not married**	30,842 (33.7%)	15,806 (36.1%)	0.051	46,665 (34.5%)	46,672 (34.4%)	-0.002
**Unknown**	24,354 (26.6%)	9,182 (21.0%)	-0.132	33,516 (24.8%)	33,887 (25.0%)	0.005
**Yost Quintile** [d], **N (Col %)**						
**Q1 (Lowest SES)**	11,356 (12.4%)	6,543 (14.9%)	0.074	17,859 (13.2%)	17,844 (13.2%)	-0.001
**Q2**	13,428 (14.7%)	6,706 (15.3%)	0.019	20,092 (14.8%)	20,059 (14.8%)	-0.002
**Q3**	15,254 (16.6%)	7,837 (17.9%)	0.033	23,047 (17.0%)	22,848 (16.8%)	-0.005

© 2026 Hu X et al. *JAMA Network Open*.

Variable	Before Weighting			Post Weighting		
	TM	MA	SMD	TM	MA	SMD
**Q4**	17,694 (19.3%)	9,325 (21.3%)	0.050	27,048 (20.0%)	27,074 (20.0%)	-0.001
**Q5 (Highest SES)**	22,962 (25.1%)	10,076 (23.0%)	-0.048	33,088 (24.4%)	33,222 (24.5%)	0.001
**Unknown**	10,934 (11.9%)	3,287 (7.5%)	-0.15	14,215 (10.5%)	14,618 (10.8%)	0.009
**Cancer site, N (Col %)**						
**Breast**	6,422 (7.0%)	2,815 (6.4%)	-0.023	9,236 (6.8%)	9,188 (6.8%)	-0.002
**Colorectal**	12,953 (14.1%)	5,908 (13.5%)	-0.019	18,881 (14.0%)	18,928 (14.0%)	0.000
**NSCLC**	40,409 (44.1%)	19,567 (44.7%)	0.012	59,911 (44.3%)	60,044 (44.3%)	0.000
**Pancreas**	11,328 (12.4%)	5,509 (12.6%)	0.007	16,841 (12.4%)	17,034 (12.6%)	0.003
**Prostate**	11,668 (12.7%)	5,852 (13.4%)	0.019	17,495 (12.9%)	17,428 (12.8%)	-0.002
**SCLC**	8,848 (9.7%)	4,123 (9.4%)	-0.008	12,984 (9.6%)	13,043 (9.6%)	0.001
**NCI Comorbidity Index, Mean (SD)**	0.53 (0.60)	0.58 (0.60)	0.095	0.54 (0.75)	0.54 (1.02)	0.000

* *Notes*: HMO = Health Maintenance Organization; MA=Medicare Advantage; NSCL = Non-Small Cell Lung; SCL = Small Cell Lung; PPO = Preferred Provider Organization; POS = Point of Service; SES = Socioeconomic Status; TM=Traditional Medicare.

[a] Other group includes American Indian/Alaska Native, Asian/Pacific Islander, and other unspecified.

[b] Based on 2013 Rural Urban Continuum Code. Codes 1-3 were classified as metro, and Codes 4-9 were classified as non-metro.

[c] With state buy-in (Medicaid) from cancer diagnosis to the end of follow-up.

[d] Yost index is a composite SES scores for census tracts based on Median household income, Median house value, Median rent, Percent below 150% of federal poverty line, Education Index, Percent working class, and Percent unemployed. Lower quintiles represent the lower SES groups.

© 2026 Hu X et al. *JAMA Network Open*.

eTable 14. Palliative Care, Systemic Therapy, and Hospice Enrollment Comparison Between Medicare Advantage and Traditional Medicare With and Without Controlling for Treating Oncologist Fixed Effects Using Propensity Score Weighted Sample

Outcome	Variable	No Physician Fixed Effects		With Physician Fixed Effects	
		HR (95%CI)	P-value	HR (95%CI)	P-value
Palliative Care[a]	**Medicare Plan Type**				
	**TM**	Ref		Ref	
	**MA**	1.38 (1.33, 1.43)	<.001	1.08 (1.03, 1.13)	0.001
Systemic Therapy[b]	**Medicare Plan Type**				
	**TM**	Ref		Ref	
	**MA**	-5.31 (-5.97, -4.64)	<.001	-5.99 (-6.80, -5.17)	<.001
Hospice Enrollment[b]	**Medicare Plan Type**				
	**TM**	Ref		Ref	
	**MA**	3.35 (2.75, 3.95)	<.001	2.07 (1.21, 2.93)	<.001

* *Notes*: MA=Medicare Advantage; TM=Traditional Medicare. All models used propensity score weighted sample for beneficiary level characteristics including age, sex, race and ethnicity, metropolitan residency, dual eligibility, marital status, Yost quintile, cancer site, year of diagnosis, and NCI comorbidity index.

[a] Treating oncologist fixed effects were implemented through fitting different baseline hazard function for different treating oncologists (i.e., treating oncologist specific baseline hazard) under Fine-Gray subdistribution hazard model.

[b] Treating oncologist fixed effects were implemented using physician dummy variables under multivariable linear probability model.

© 2026 Hu X et al. *JAMA Network Open*.
