# Supplementary material for: Palliative Care for Advanced Cancer in Medicare Advantage and Physician Networks
Source: JAMA Netw Open. 2026 Sep 30;9(9):e2636865. doi: 10.1001/jamanetworkopen.2026.36865 (PMC13628318; doi:10.1001/jamanetworkopen.2026.36865)
Supplement: Supplementary file 2 [file jamanetwopen-e2636865-s002.pdf]

# Data Sharing Statement

Hu. Palliative Care for Advanced Cancer in Medicare Advantage and Traditional Medicare Physician Networks. *JAMA Netw Open*. Published September 30, 2026.
doi:10.1001/jamanetworkopen.2026.36865

**Data**

**Data available:** The dataset used to conduct this study is available upon approval of a research protocol from the National Cancer Institute. Instructions for obtaining these data are available at https://healthcaredelivery.cancer.gov/seermedicare/obtain/.
